# Supplementary material for: ZAP targets aberrant mRNA transcripts encoding proteins with defective signal peptides for degradation
Source: EMBO J. 2026 Mar 12;45(8):2638–65. doi: 10.1038/s44318-026-00720-4 (PMC13084044; doi:10.1038/s44318-026-00720-4)
Supplement: Supplementary file 1 — Appendix [file 44318_2026_720_MOESM1_ESM.pdf]

Appendix for:

ZAP targets aberrant mRNA transcripts encoding proteins with defective signal peptides for degradation

Table of contents

Appendix Figure S1 ……………………………………………………………………………………………………page 2

Appendix Figure S2 ……………………………………………………………………………………………………page 3

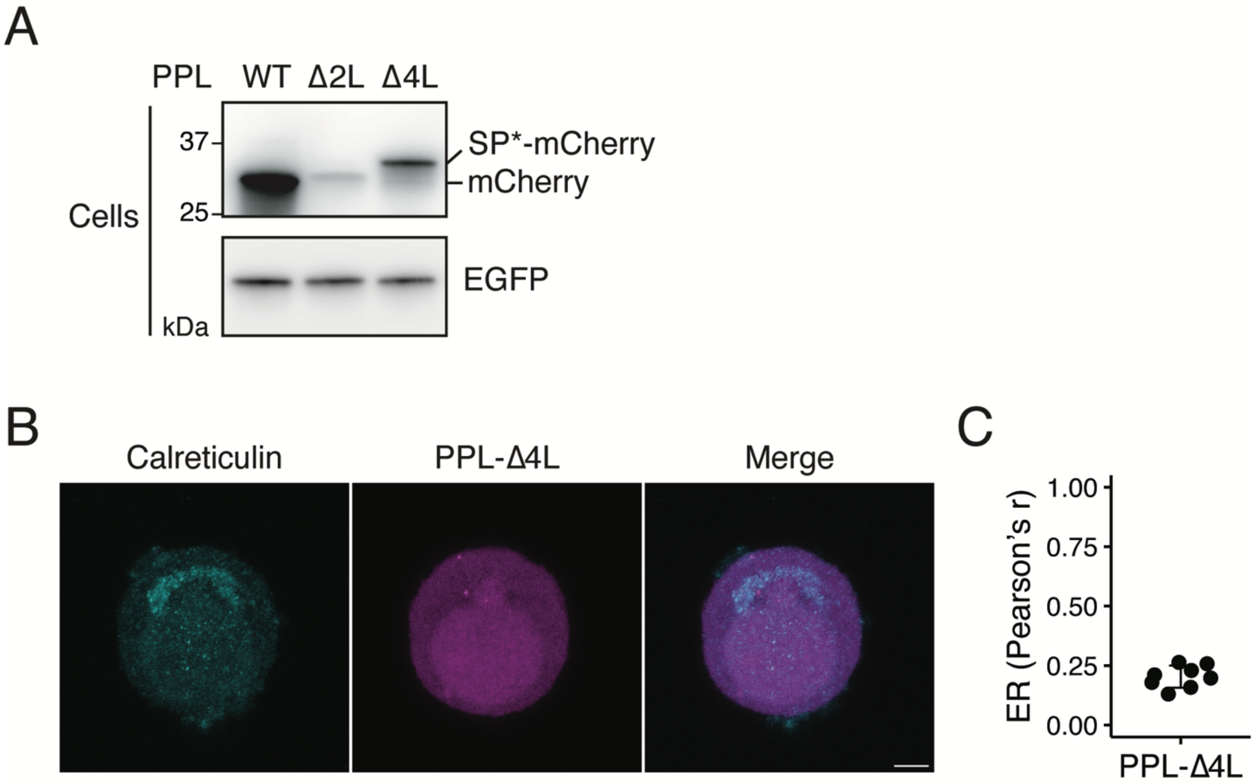

Appendix Figure S1. Δ4L mutation in the PPL signal peptide reduces cleavage efficiency

(A) Immunoblots for the PPL reporters expressed under a constitutive SFFV promoter. EGFP serves as an expression and loading control. SP*-mCherry denotes the reporter with an uncleaved signal peptide.

(B) Representative images of K562 cells expressing the SFFV-driven PPL-Δ4L reporter (A), stained with anti-Calreticulin and anti-FLAG (PPL-Δ4L reporter) antibodies.

(C) Quantification of PPL-Δ4L colocalization with the calreticulin-positive pixels using Pearson's r values. Error bar indicates standard deviations (n= 8).

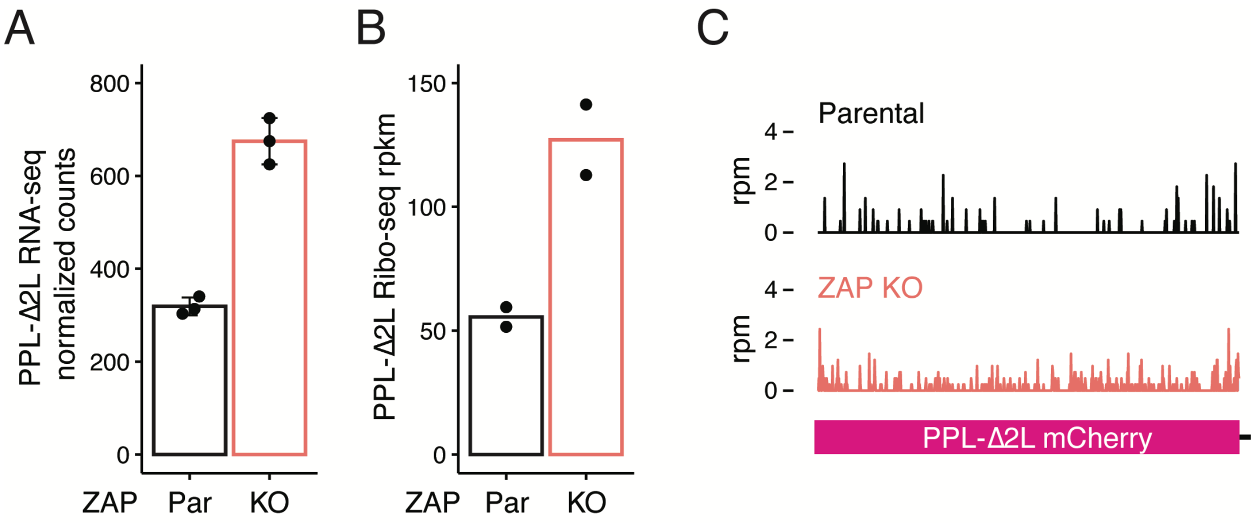

Appendix Figure S2. ZAP is not required for RAPP-mediated translational repression

(A) Normalized counts for the PPL-Δ2L reporter mRNA from the RNA-seq experiments. Error bars indicate standard deviations (biological replicates, n= 3).

(B) Ribosome footprint occupancy for the PPL-Δ2L reporter mRNA (biological replicates, n= 2).

(C) Ribosome footprints across the PPL-Δ2L reporter mRNA in parental and ZAP KO cells.
