# Supplementary figures and images for: ZAP targets aberrant mRNA transcripts encoding proteins with defective signal peptides for degradation
Source: EMBO J. 2026 Mar 12;45(8):2638–65. doi: 10.1038/s44318-026-00720-4 (PMC13084044; doi:10.1038/s44318-026-00720-4)

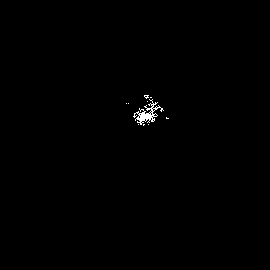

Supplement: Supplementary file 6 — Source data Fig. 1 [file 44318_2026_720_MOESM6_ESM.zip › SDfigure1/1D/Mask.tif]

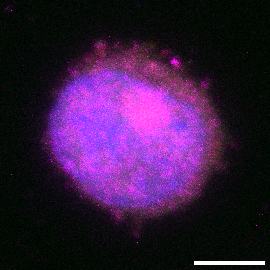

Supplement: Supplementary file 6 — Source data Fig. 1 [file 44318_2026_720_MOESM6_ESM.zip › SDfigure1/1D/Merge.tif]

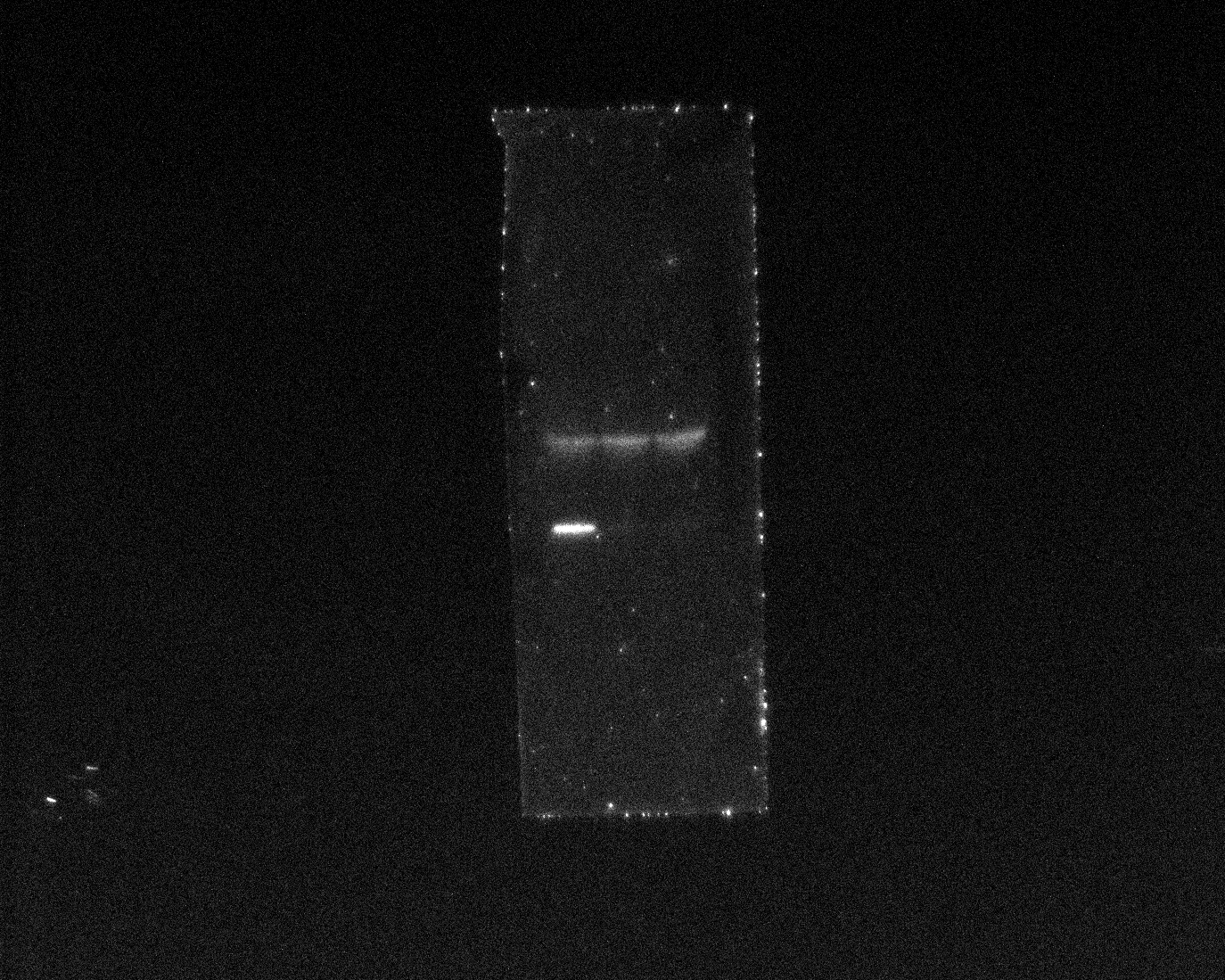

Supplement: Supplementary file 6 — Source data Fig. 1 [file 44318_2026_720_MOESM6_ESM.zip › SDfigure1/1C/media_mCherry.tif]

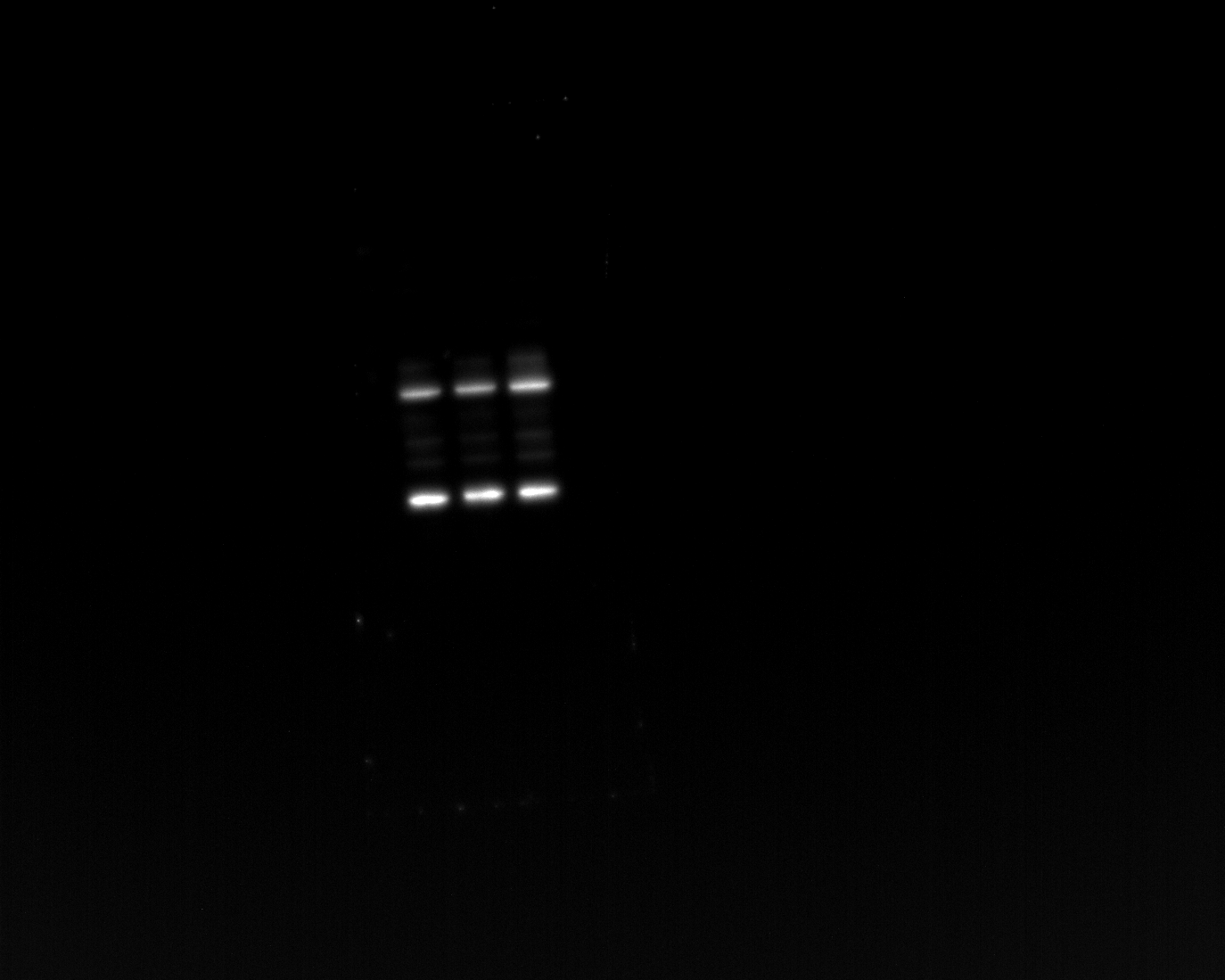

Supplement: Supplementary file 6 — Source data Fig. 1 [file 44318_2026_720_MOESM6_ESM.zip › SDfigure1/1C/cells_EGFP.tif]

cells mCherry

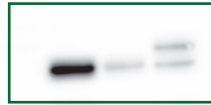

cells EGFP

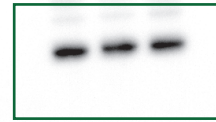

media mCherry

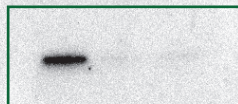

Supplement: Supplementary file 6 — Source data Fig. 1 [file 44318_2026_720_MOESM6_ESM.zip › SDfigure1/1C/README.pdf]

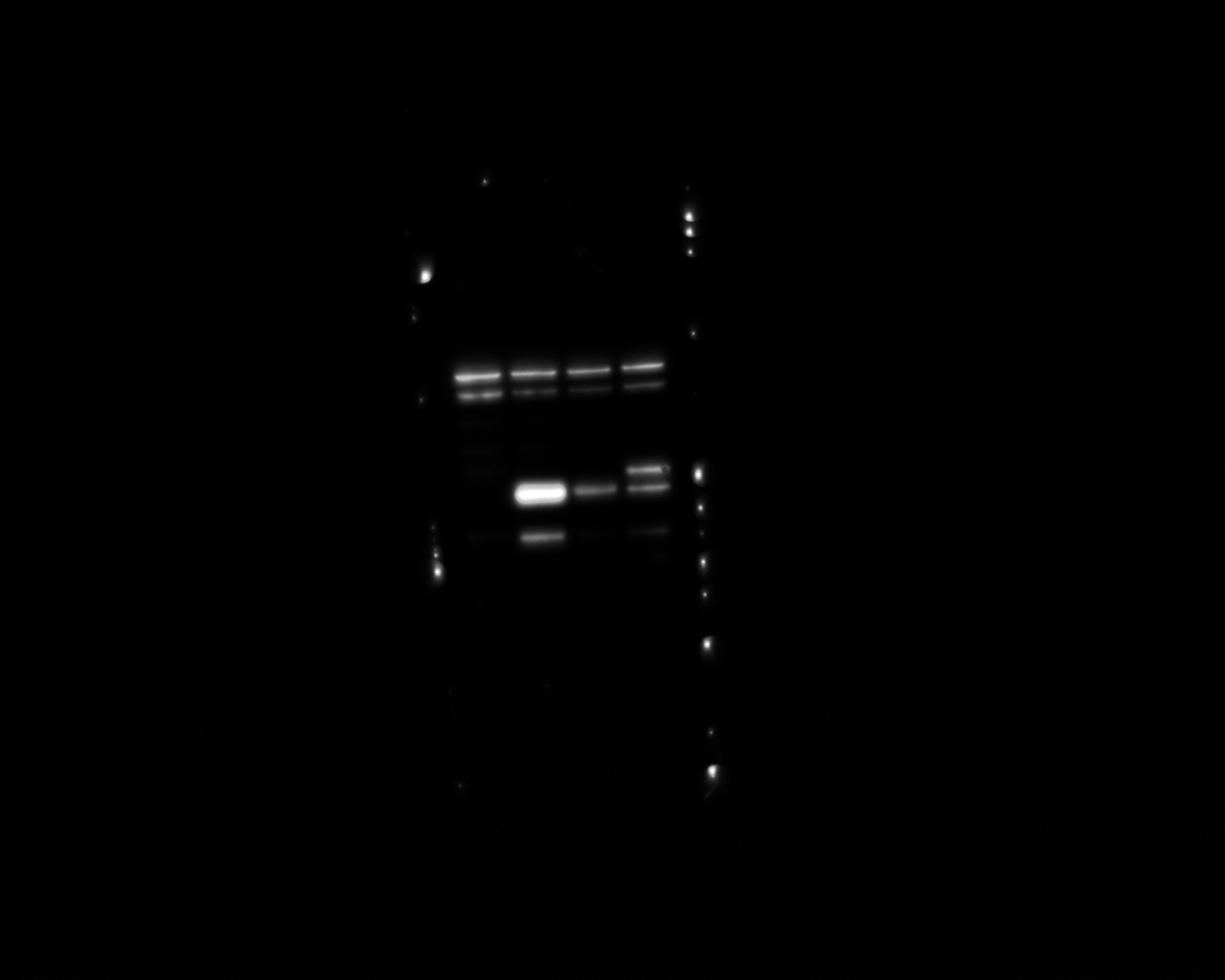

Supplement: Supplementary file 6 — Source data Fig. 1 [file 44318_2026_720_MOESM6_ESM.zip › SDfigure1/1C/cells_mCherry.tif]

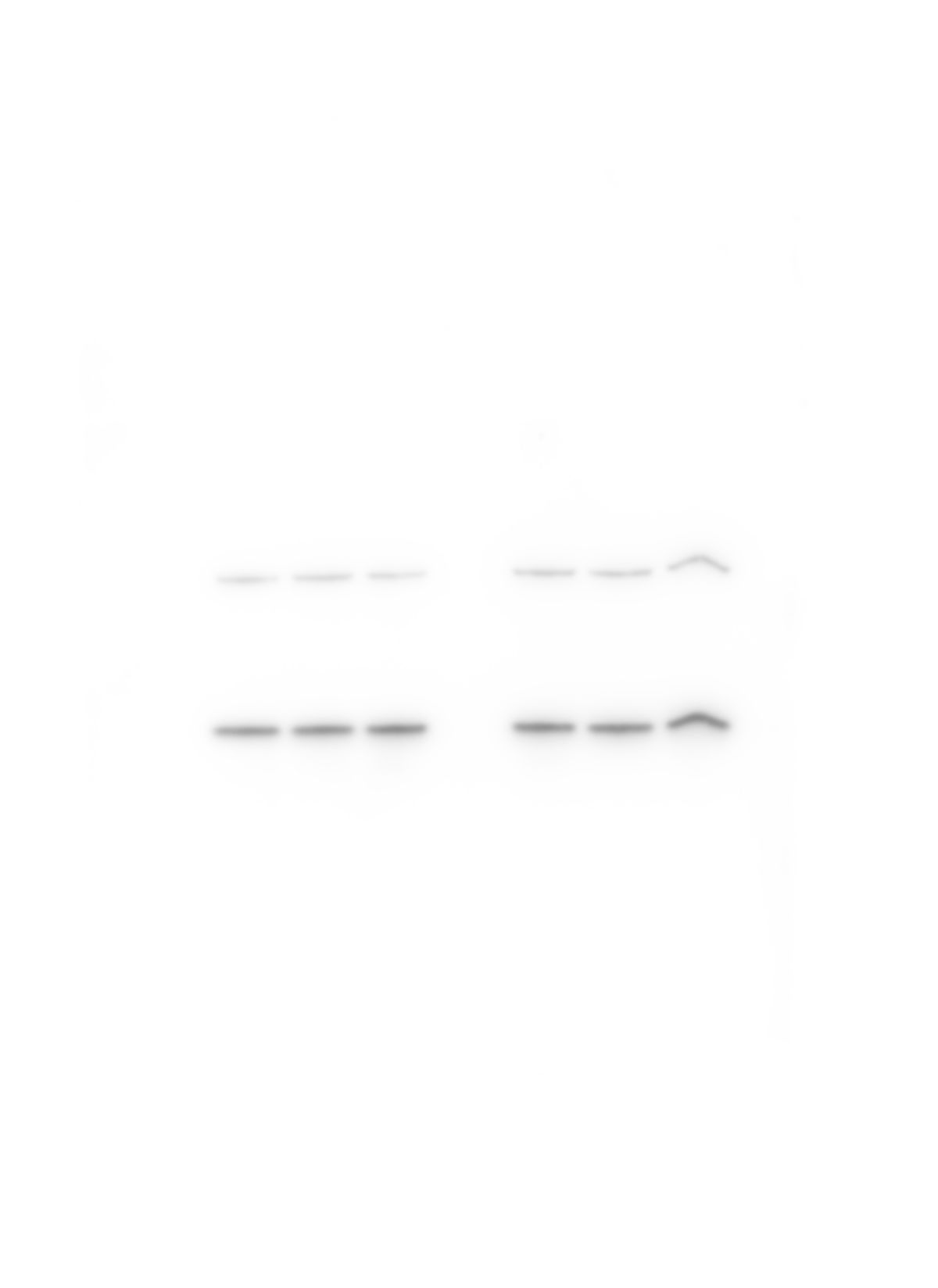

Supplement: Supplementary file 7 — Source data Fig. 2 [file 44318_2026_720_MOESM7_ESM.zip › SDfigure2/2F/EGFP.tif]

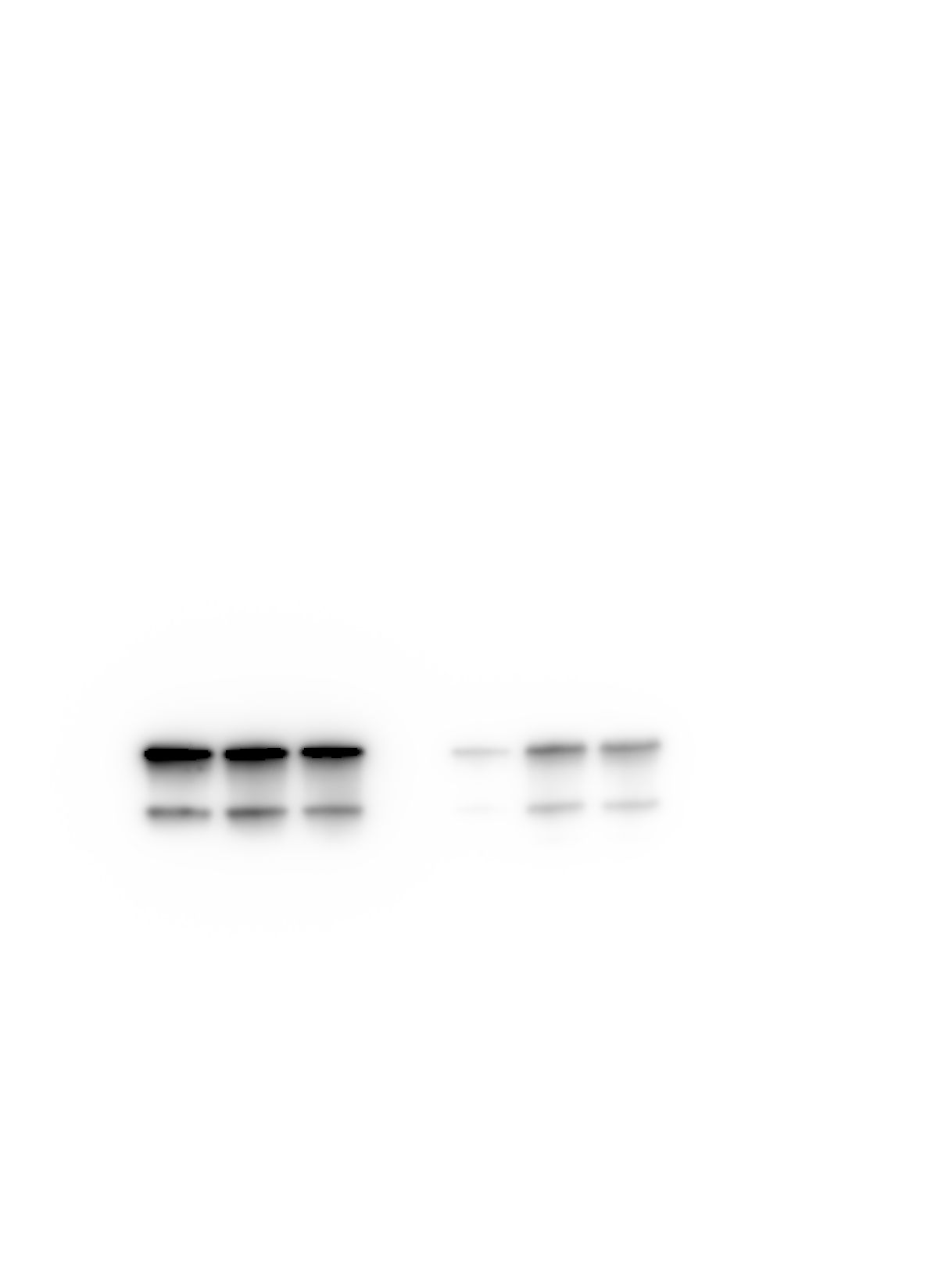

Supplement: Supplementary file 7 — Source data Fig. 2 [file 44318_2026_720_MOESM7_ESM.zip › SDfigure2/2F/mCherry.tif]

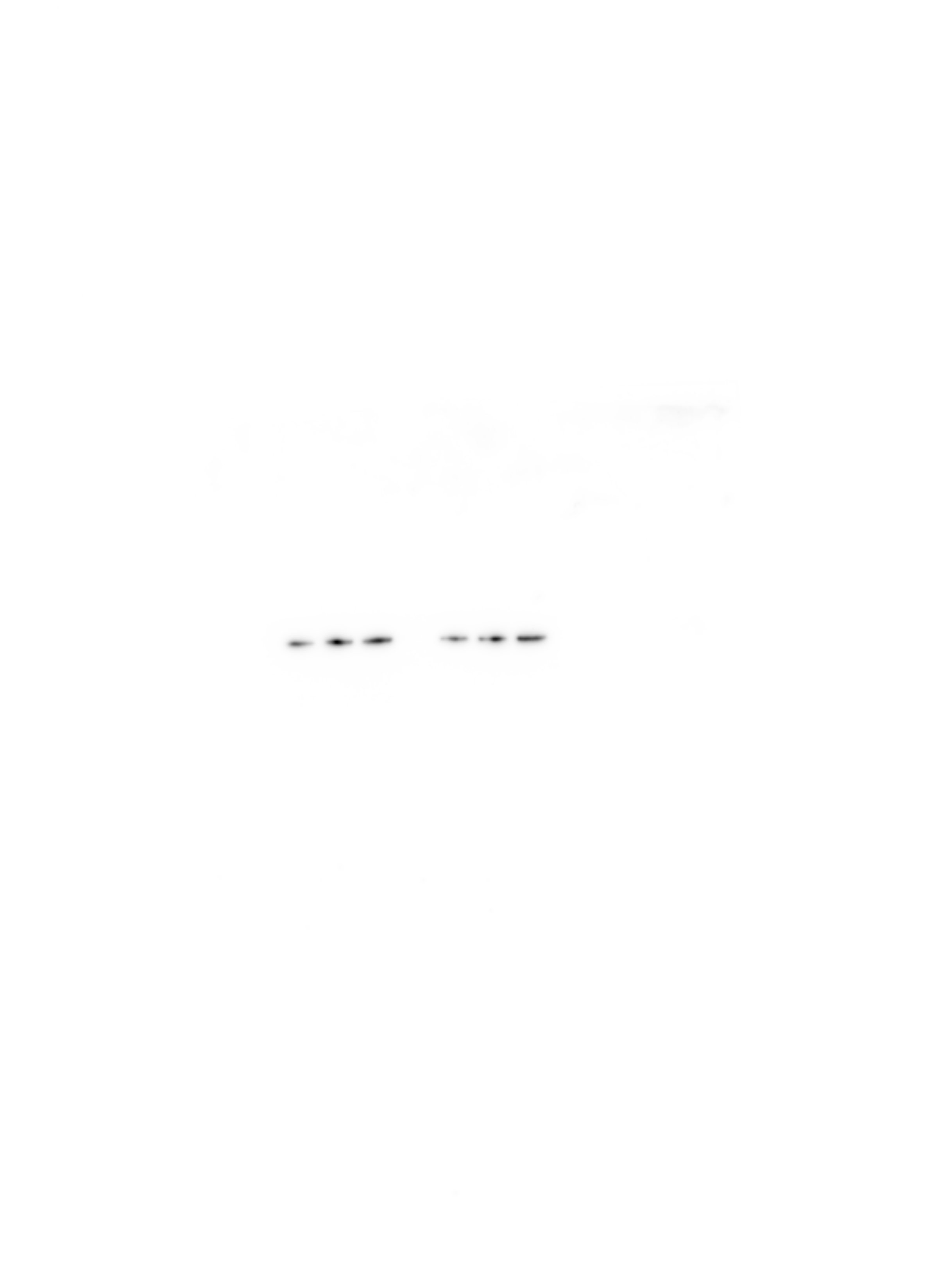

Supplement: Supplementary file 7 — Source data Fig. 2 [file 44318_2026_720_MOESM7_ESM.zip › SDfigure2/2F/bAct.tif]

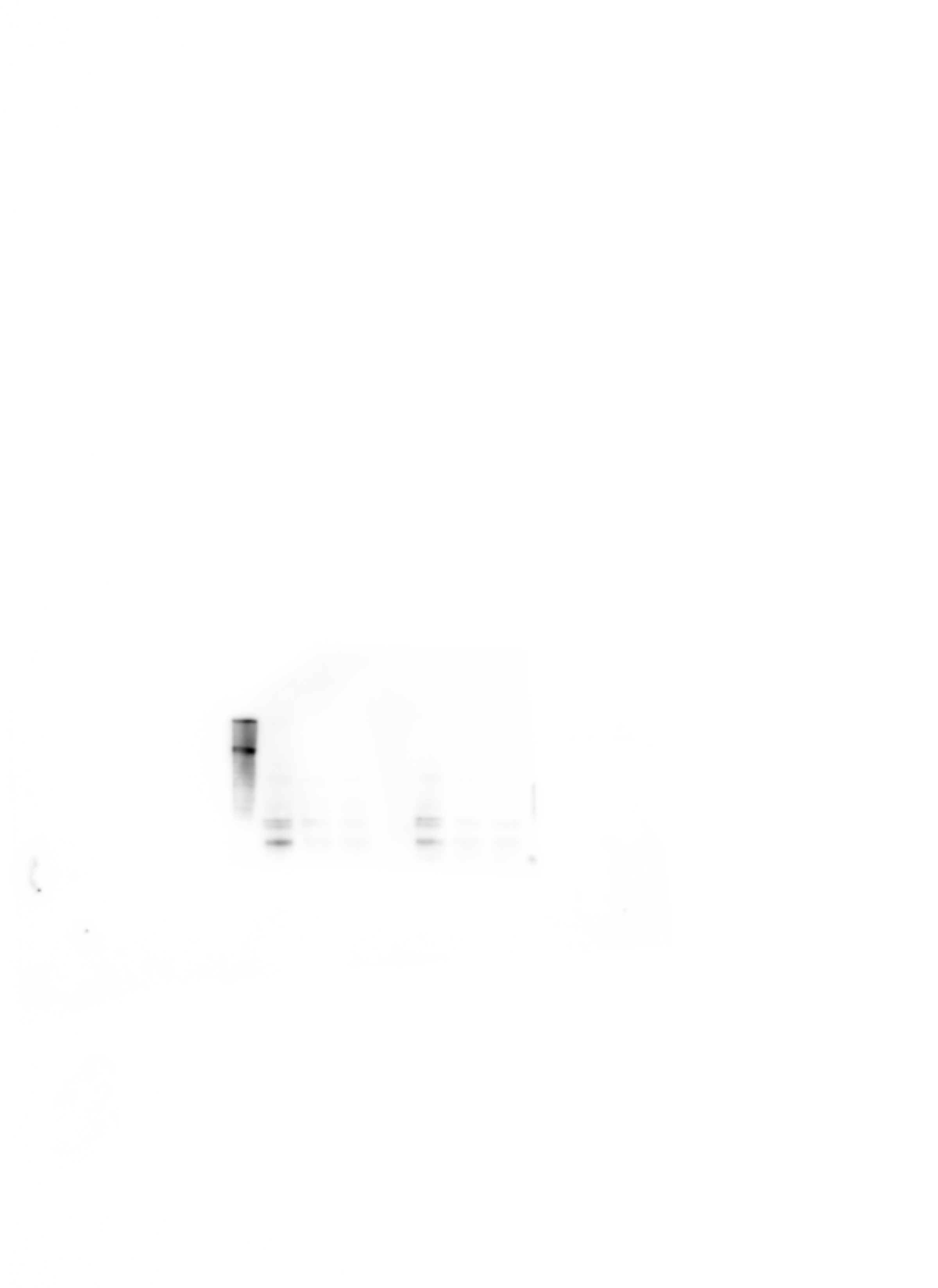

Supplement: Supplementary file 7 — Source data Fig. 2 [file 44318_2026_720_MOESM7_ESM.zip › SDfigure2/2F/ZAP.tif]

mCherry

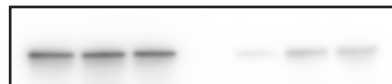

EGFP

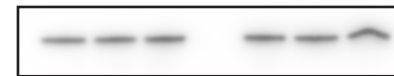

ZAP

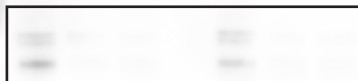

b-Act

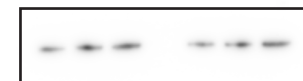

Supplement: Supplementary file 7 — Source data Fig. 2 [file 44318_2026_720_MOESM7_ESM.zip › SDfigure2/2F/README.pdf]

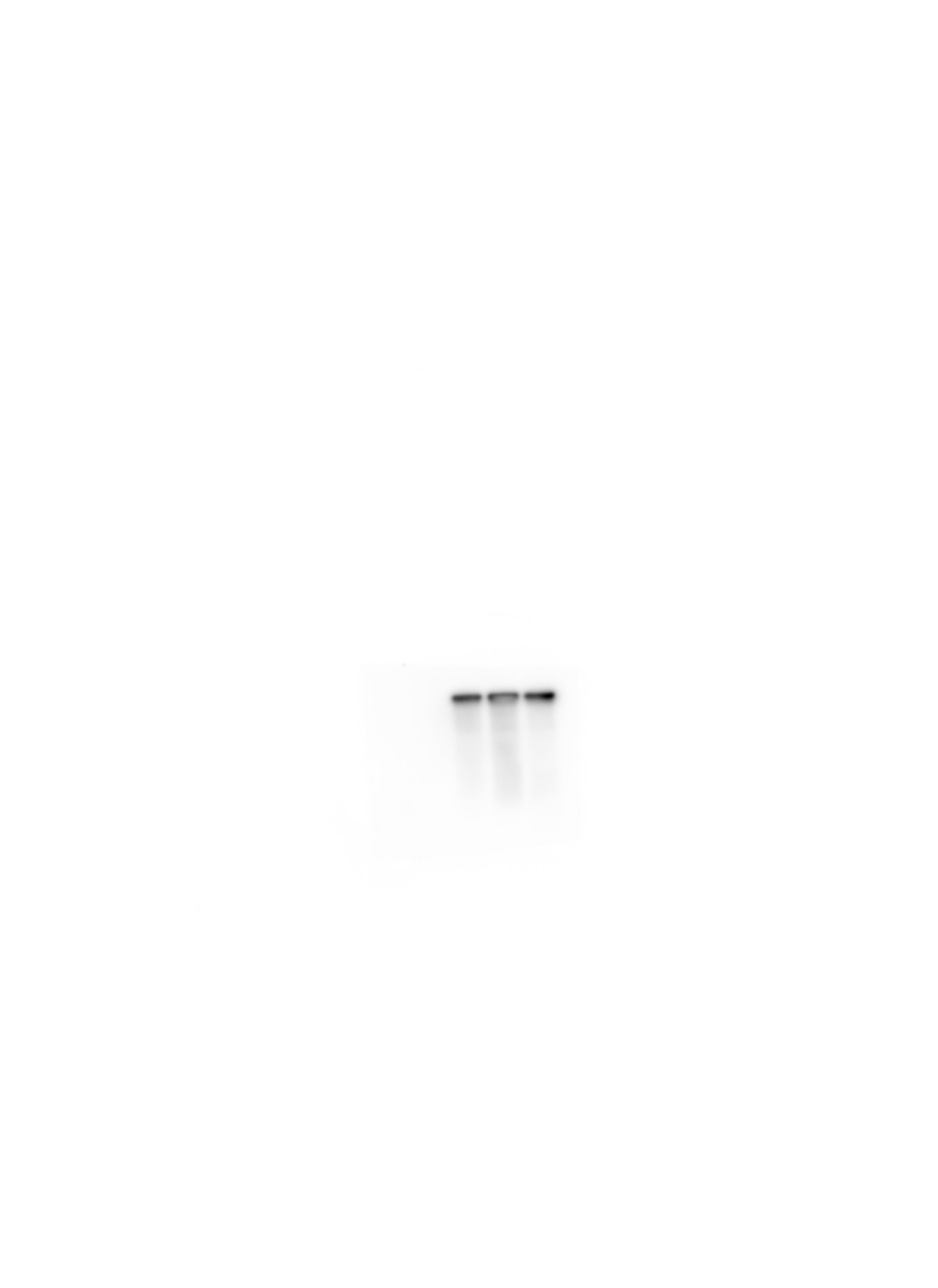

Supplement: Supplementary file 8 — Source data Fig. 3 [file 44318_2026_720_MOESM8_ESM.zip › SDfigure3/3A/EGFP.tif]

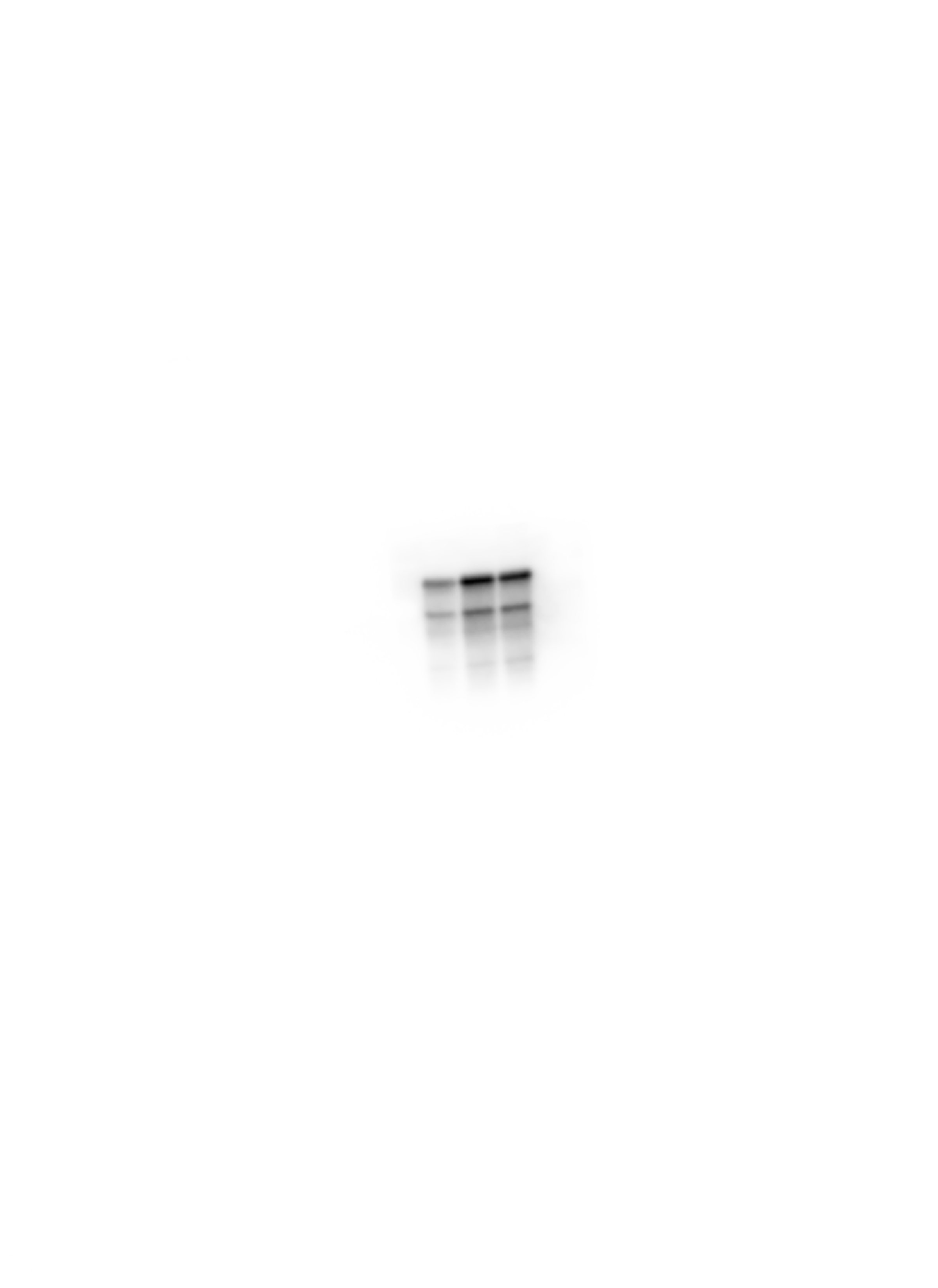

Supplement: Supplementary file 8 — Source data Fig. 3 [file 44318_2026_720_MOESM8_ESM.zip › SDfigure3/3A/mCherry.tif]

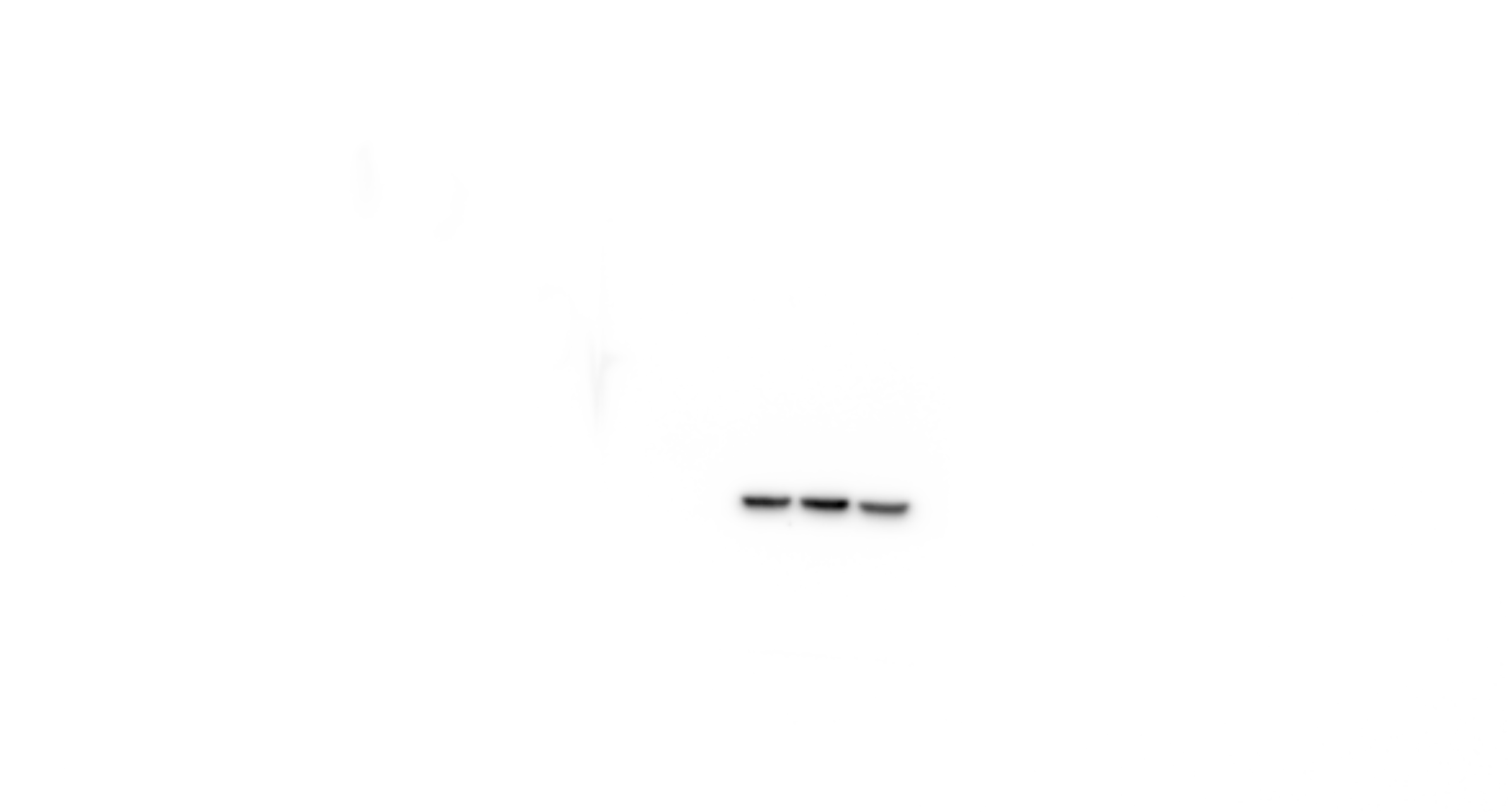

Supplement: Supplementary file 8 — Source data Fig. 3 [file 44318_2026_720_MOESM8_ESM.zip › SDfigure3/3A/bAct.tif]

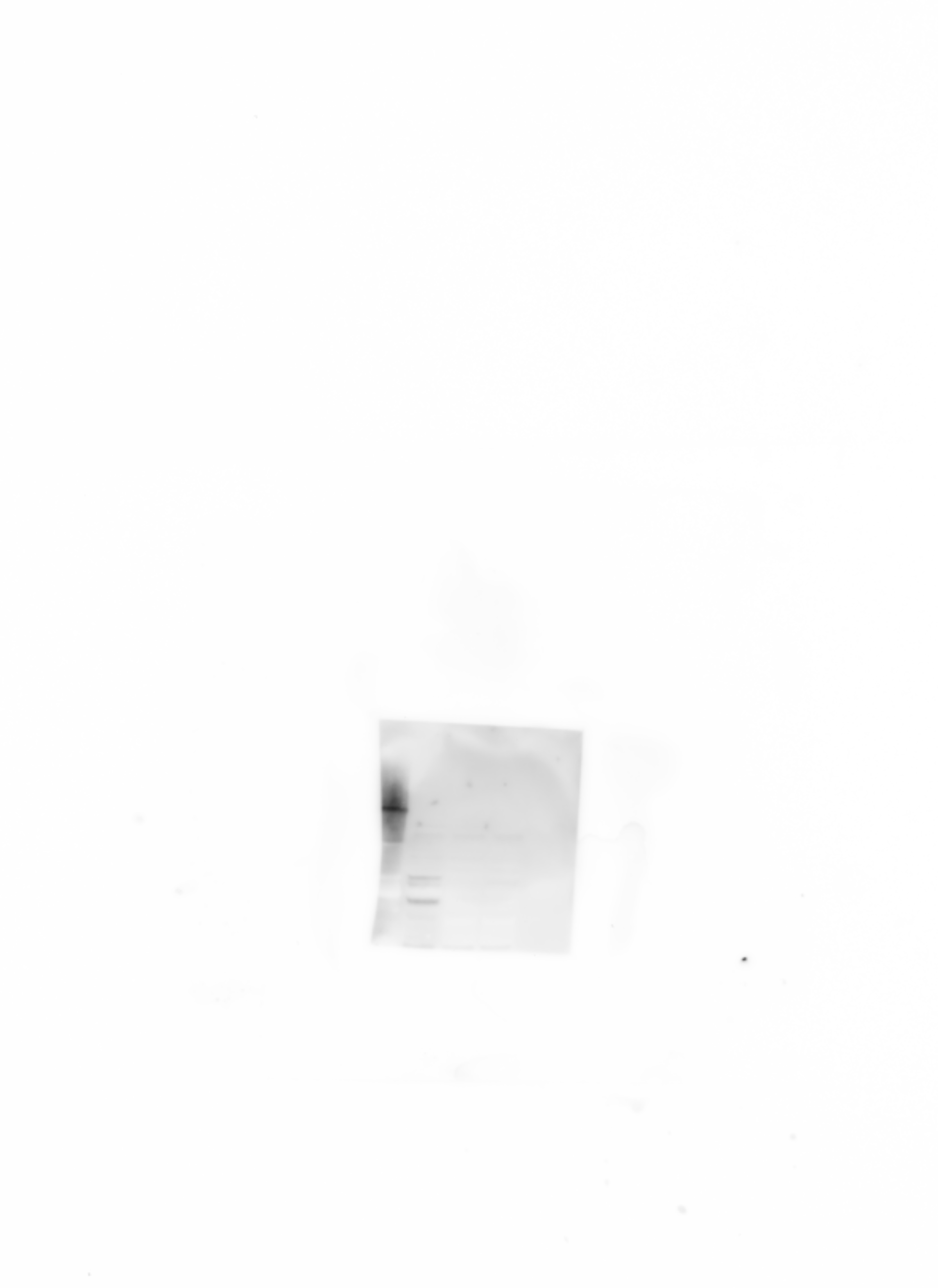

Supplement: Supplementary file 8 — Source data Fig. 3 [file 44318_2026_720_MOESM8_ESM.zip › SDfigure3/3A/ZAP.tif]

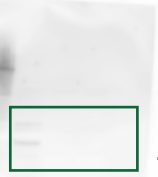

ZAP

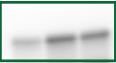

mCherry

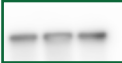

EGFP

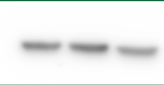

bAct

Supplement: Supplementary file 8 — Source data Fig. 3 [file 44318_2026_720_MOESM8_ESM.zip › SDfigure3/3A/README.pdf]

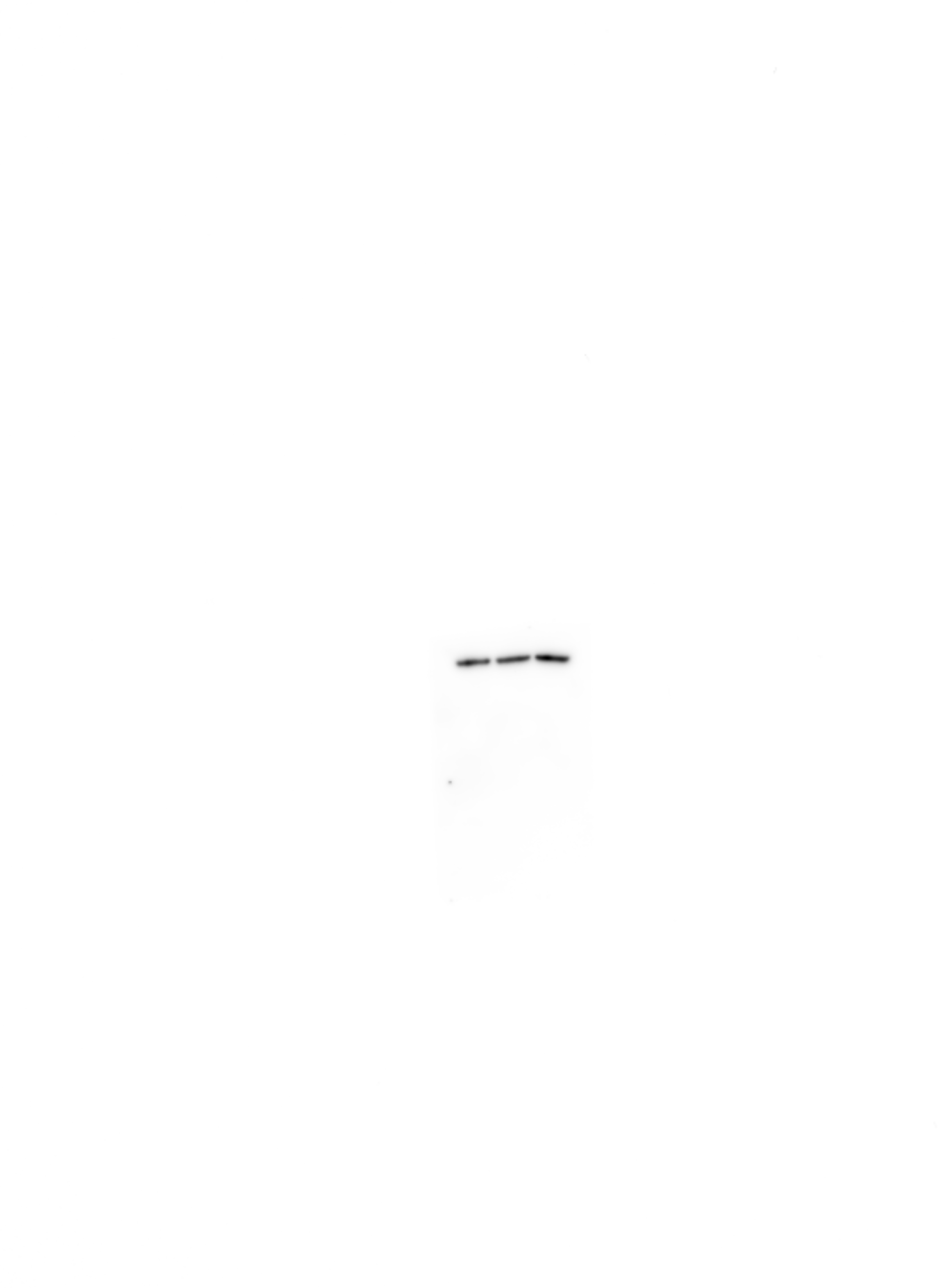

Supplement: Supplementary file 8 — Source data Fig. 3 [file 44318_2026_720_MOESM8_ESM.zip › SDfigure3/3F/bAct.tif]

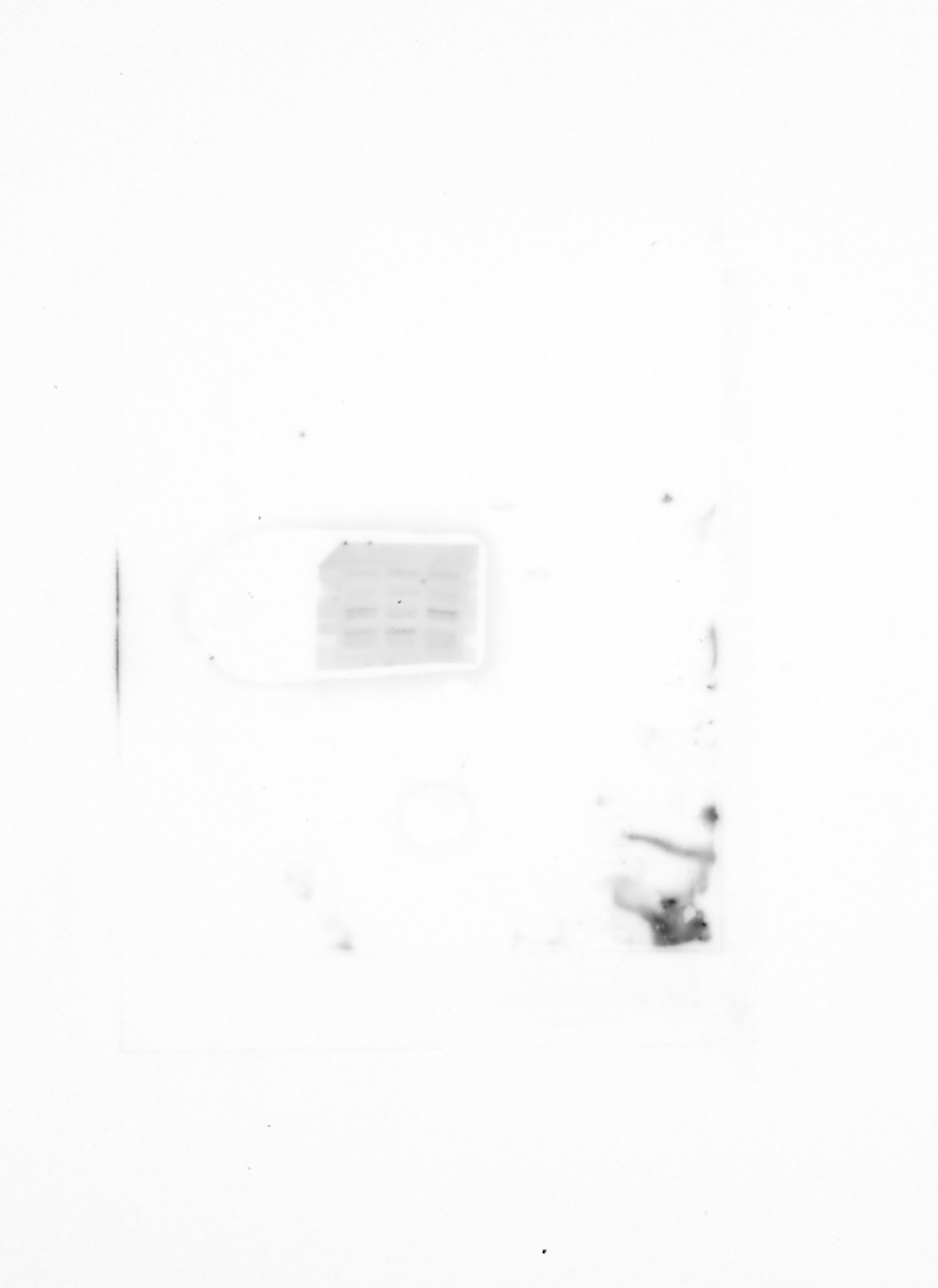

Supplement: Supplementary file 8 — Source data Fig. 3 [file 44318_2026_720_MOESM8_ESM.zip › SDfigure3/3F/ZAP.tif]

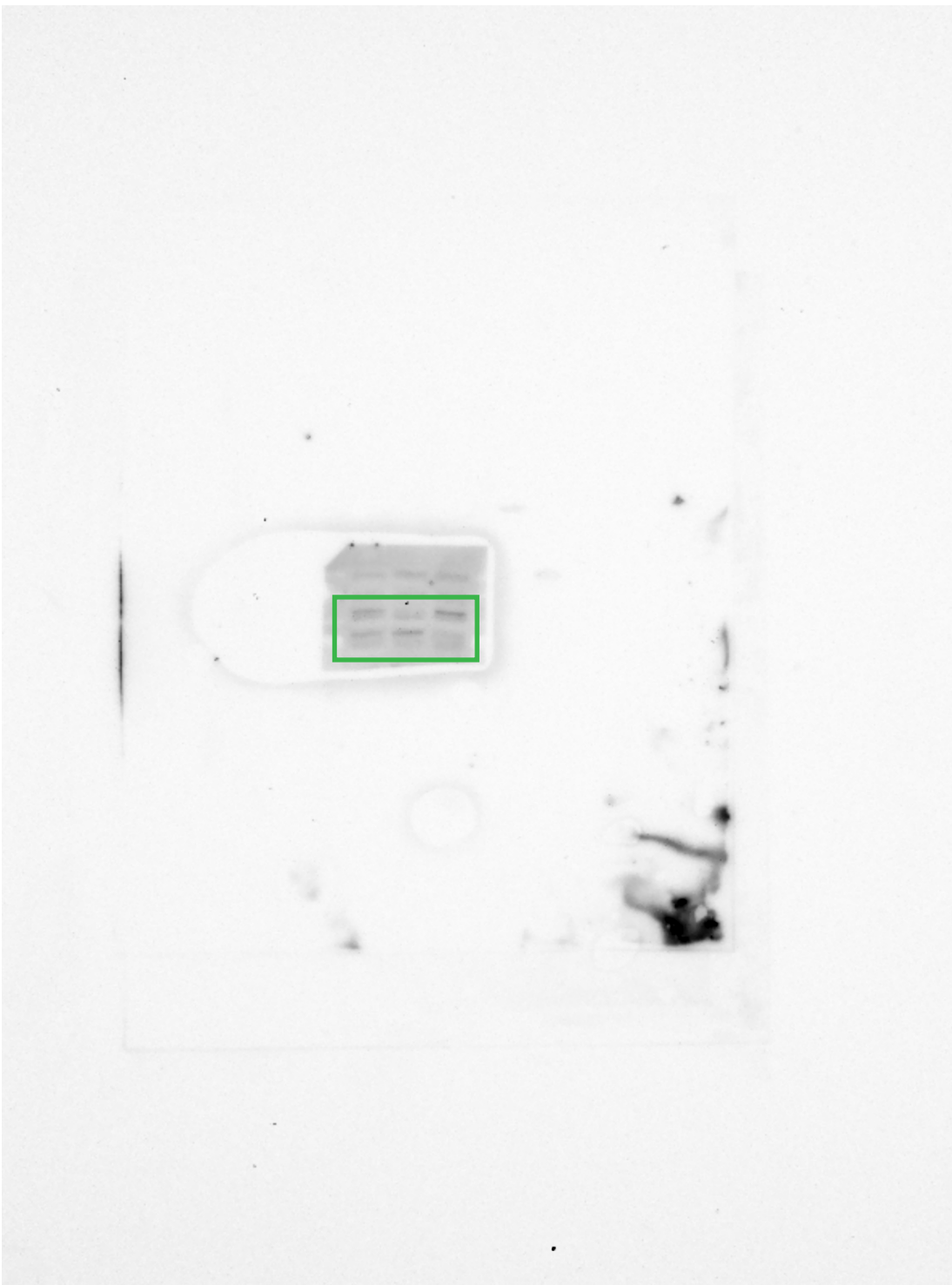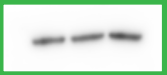

Supplement: Supplementary file 8 — Source data Fig. 3 [file 44318_2026_720_MOESM8_ESM.zip › SDfigure3/3F/README_IB.pdf]

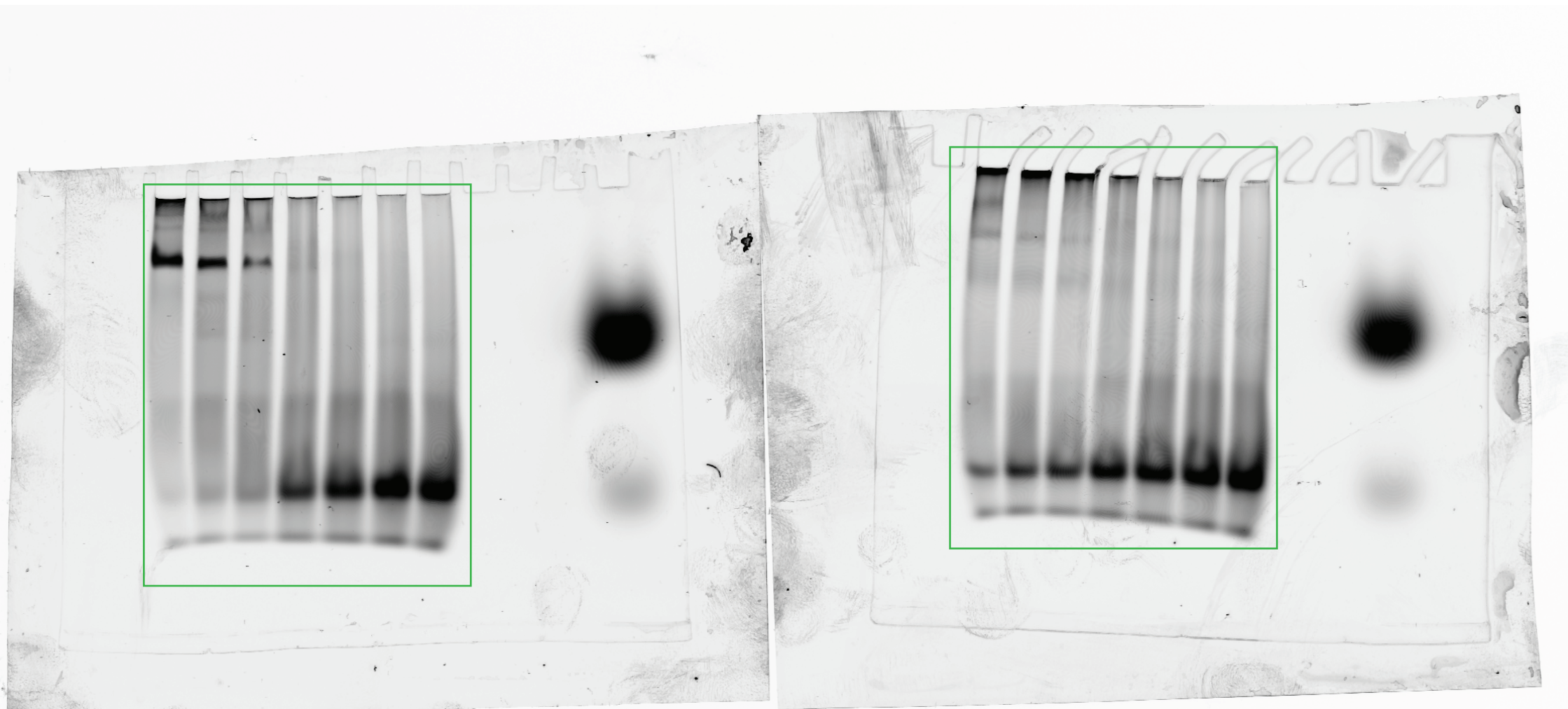

Supplement: Supplementary file 9 — Source data Fig. 4 [file 44318_2026_720_MOESM9_ESM.zip › SDfigure4/4EF/README.pdf]

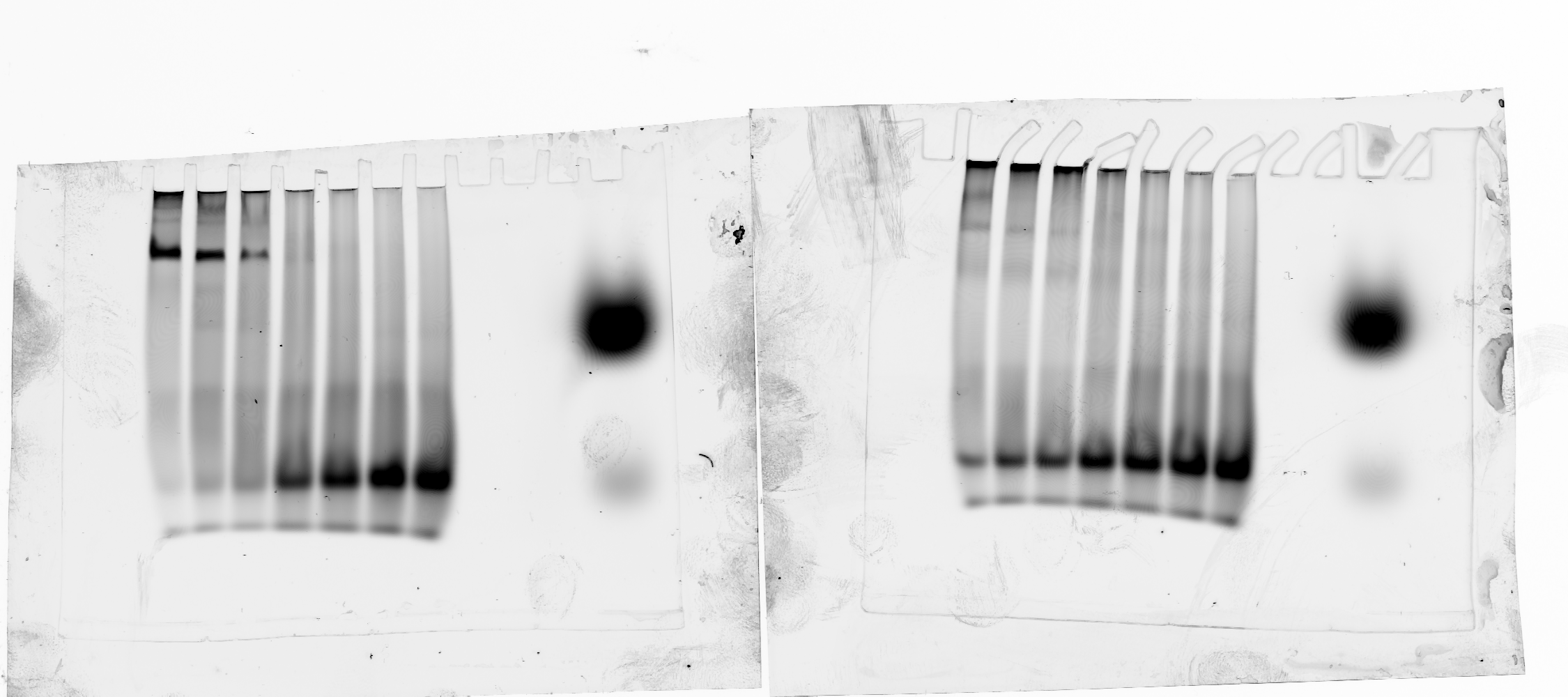

Supplement: Supplementary file 9 — Source data Fig. 4 [file 44318_2026_720_MOESM9_ESM.zip › SDfigure4/4EF/4EF.png]

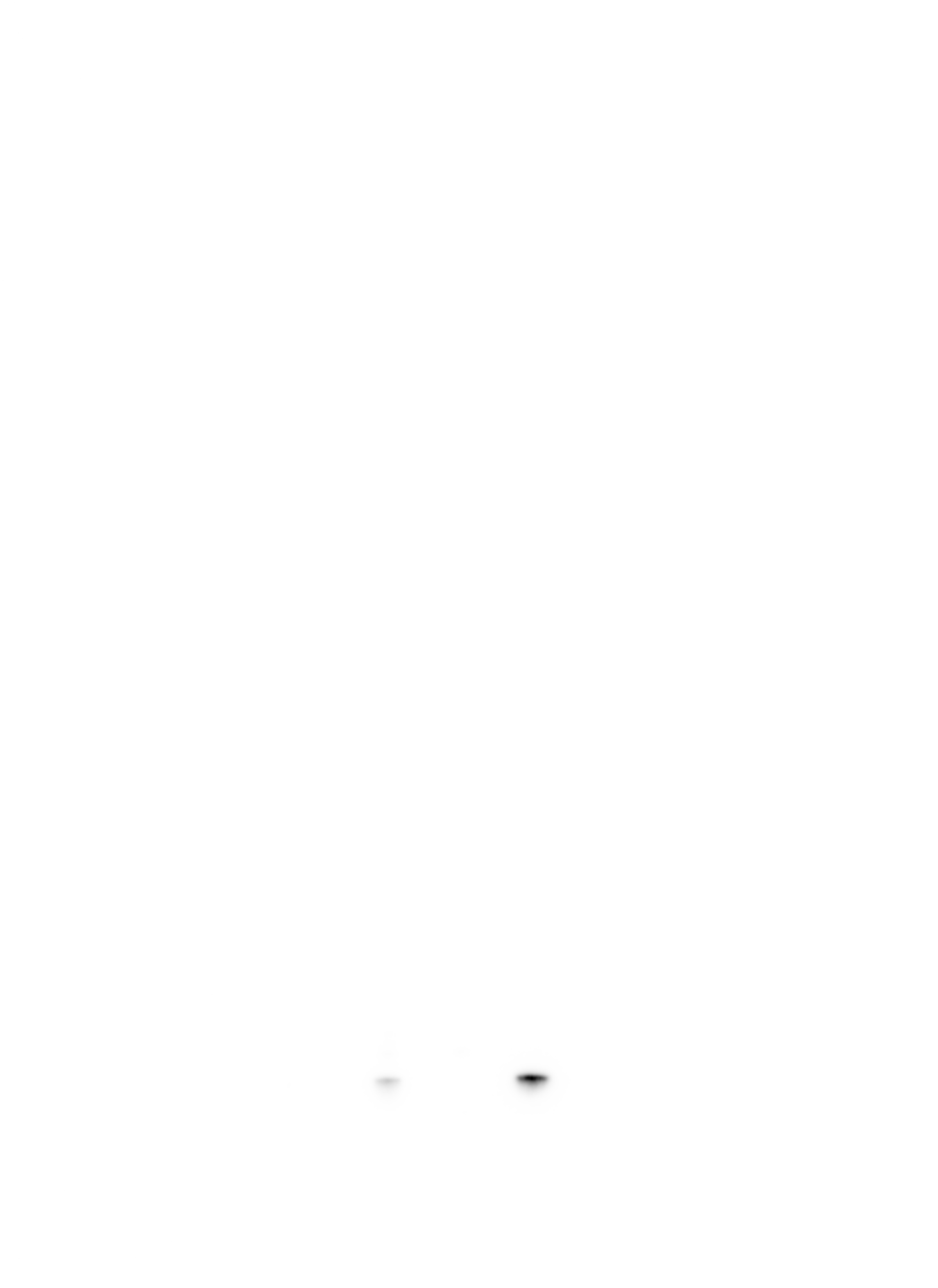

Supplement: Supplementary file 9 — Source data Fig. 4 [file 44318_2026_720_MOESM9_ESM.zip › SDfigure4/4B/SRPRB.tif]

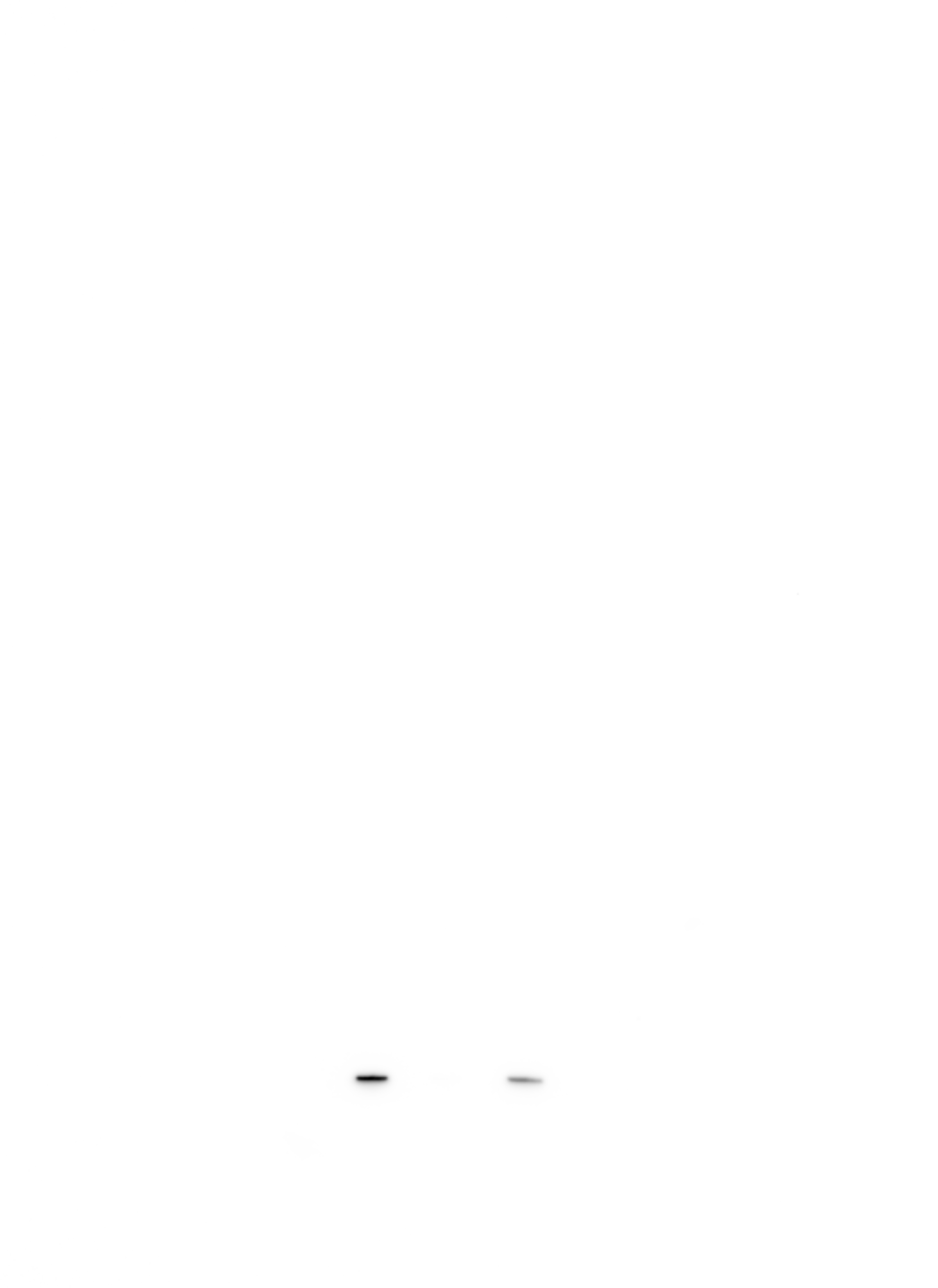

Supplement: Supplementary file 9 — Source data Fig. 4 [file 44318_2026_720_MOESM9_ESM.zip › SDfigure4/4B/calnexin.tif]

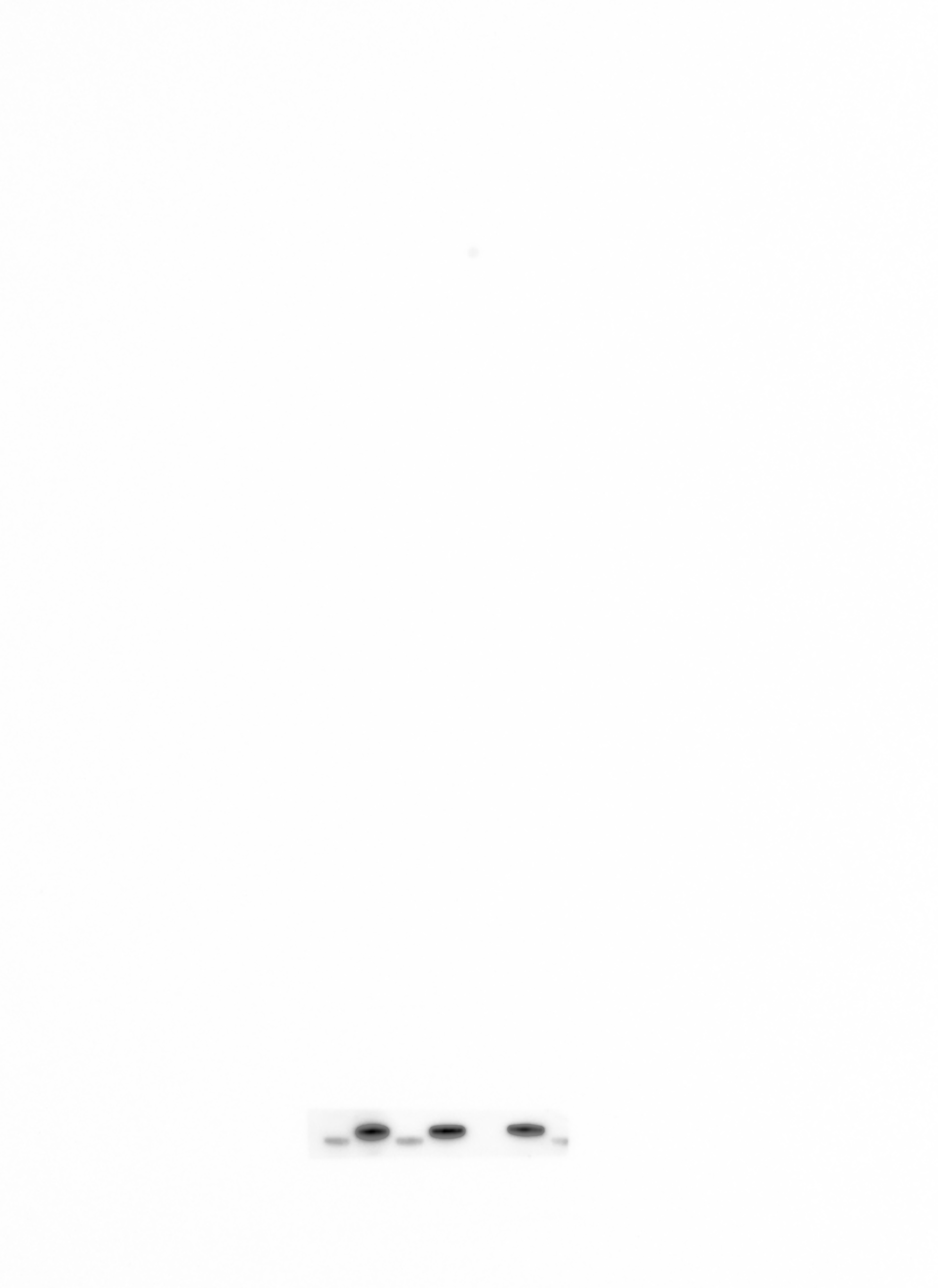

Supplement: Supplementary file 9 — Source data Fig. 4 [file 44318_2026_720_MOESM9_ESM.zip › SDfigure4/4B/ZAP-S.tif]

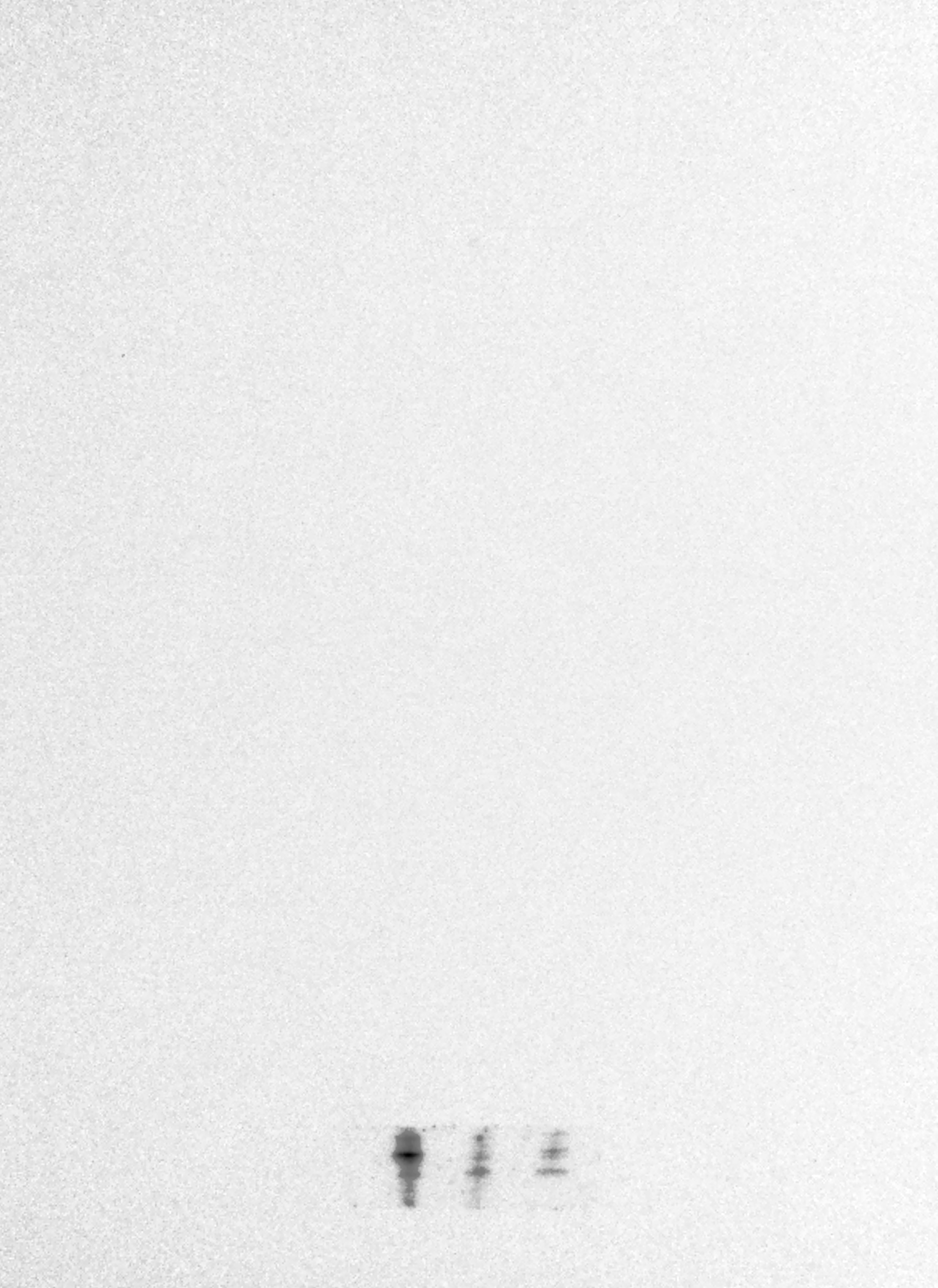

Supplement: Supplementary file 9 — Source data Fig. 4 [file 44318_2026_720_MOESM9_ESM.zip › SDfigure4/4B/VDAC.tif]

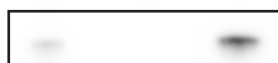

SRPRB

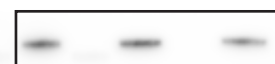

ZAP-S

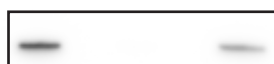

calnexin

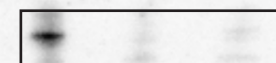

VDAC

Supplement: Supplementary file 9 — Source data Fig. 4 [file 44318_2026_720_MOESM9_ESM.zip › SDfigure4/4B/README.pdf]

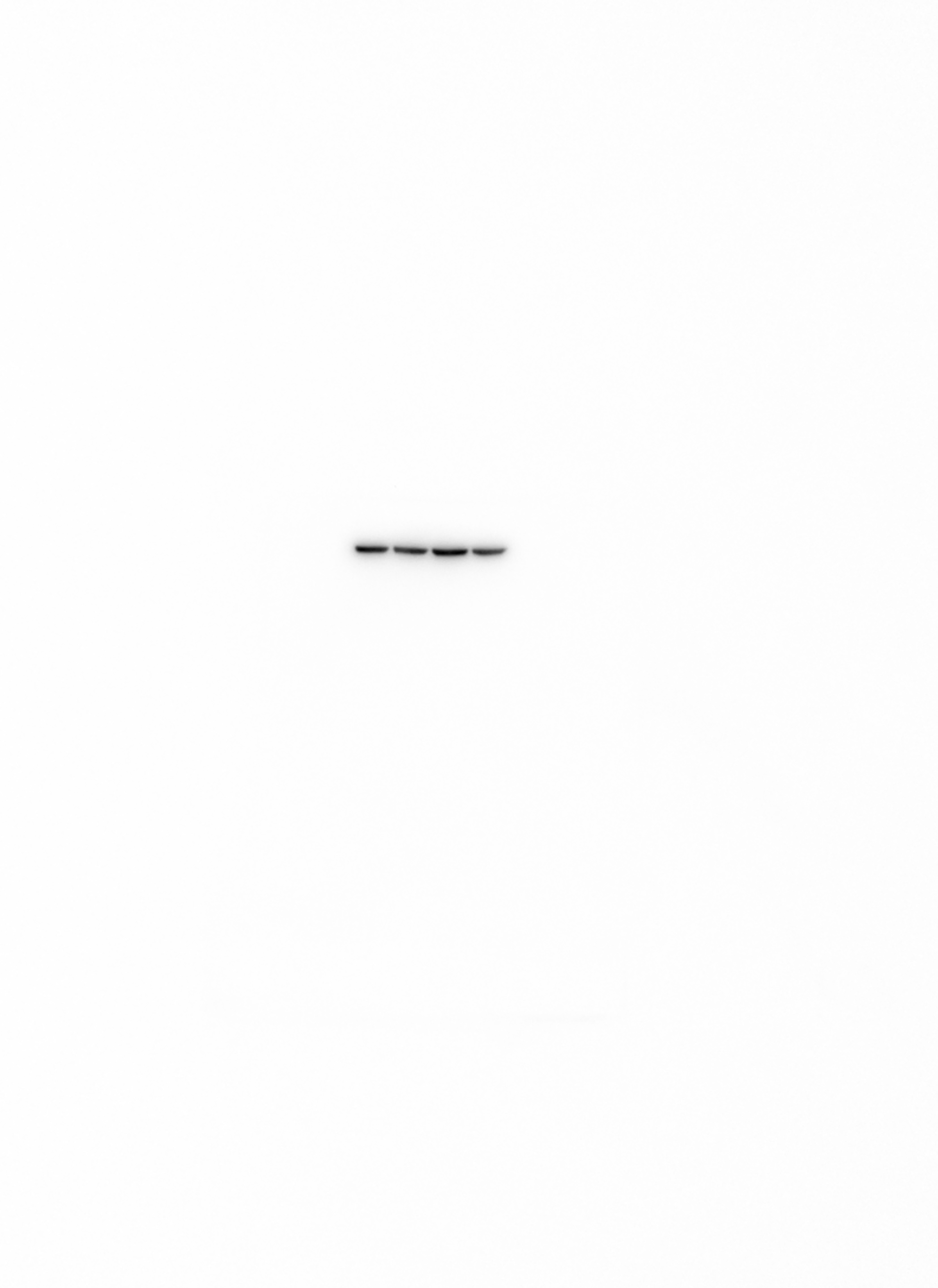

Supplement: Supplementary file 10 — Source data Fig. 5 [file 44318_2026_720_MOESM10_ESM.zip › SDfigure5/5C/bAct.tif]

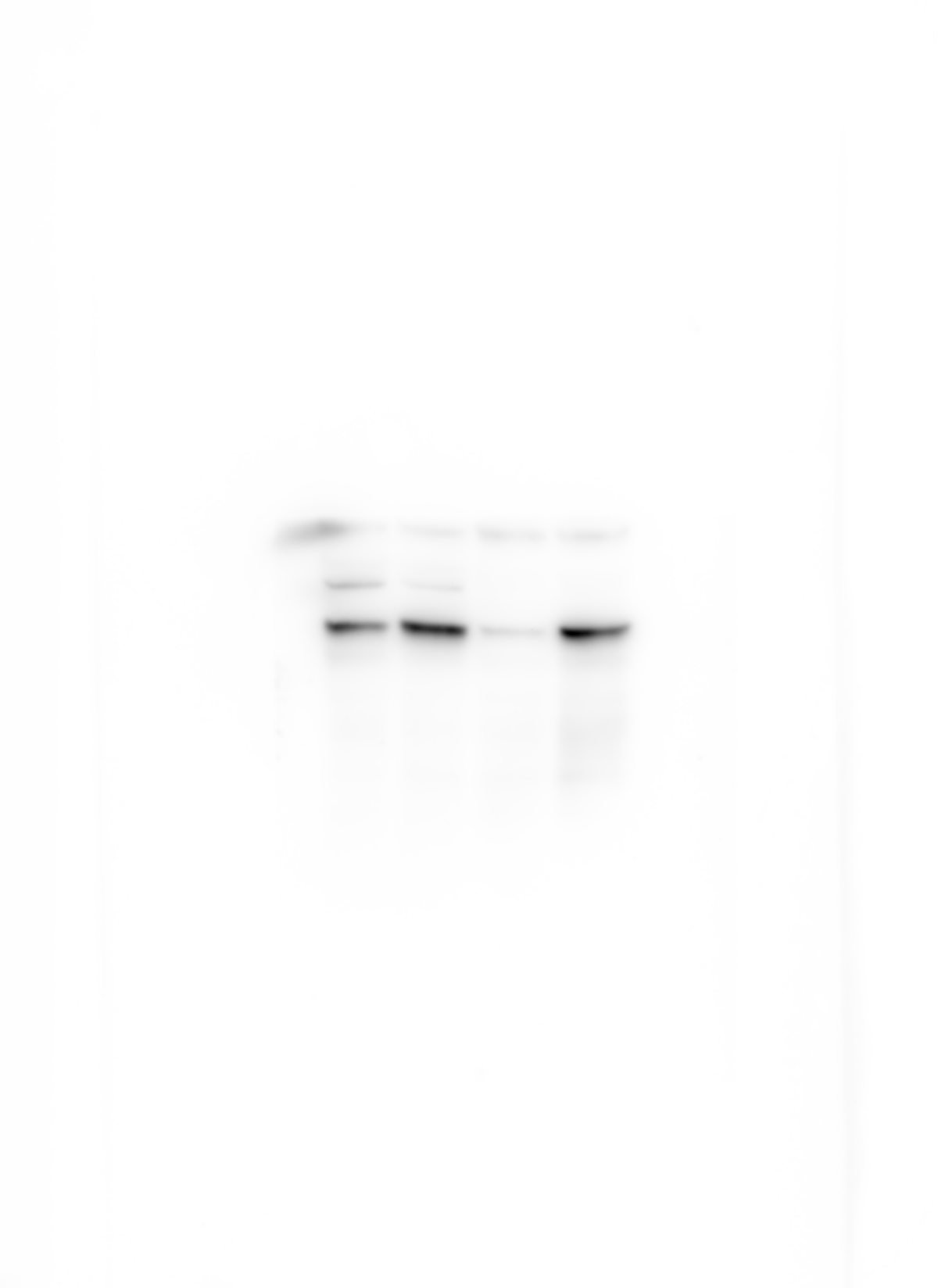

Supplement: Supplementary file 10 — Source data Fig. 5 [file 44318_2026_720_MOESM10_ESM.zip › SDfigure5/5C/eIF2aP.tif]

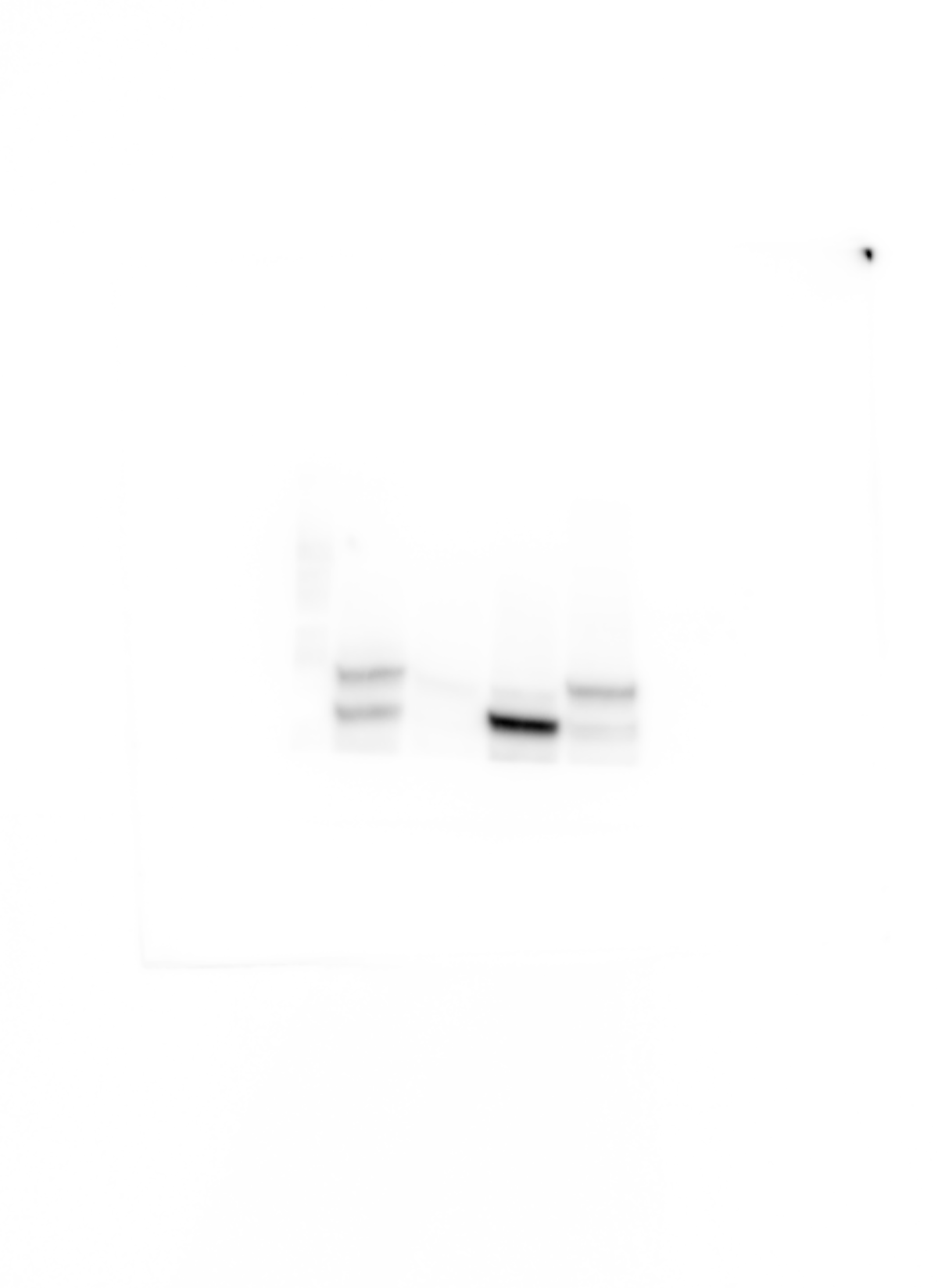

Supplement: Supplementary file 10 — Source data Fig. 5 [file 44318_2026_720_MOESM10_ESM.zip › SDfigure5/5C/ZAP.tif]

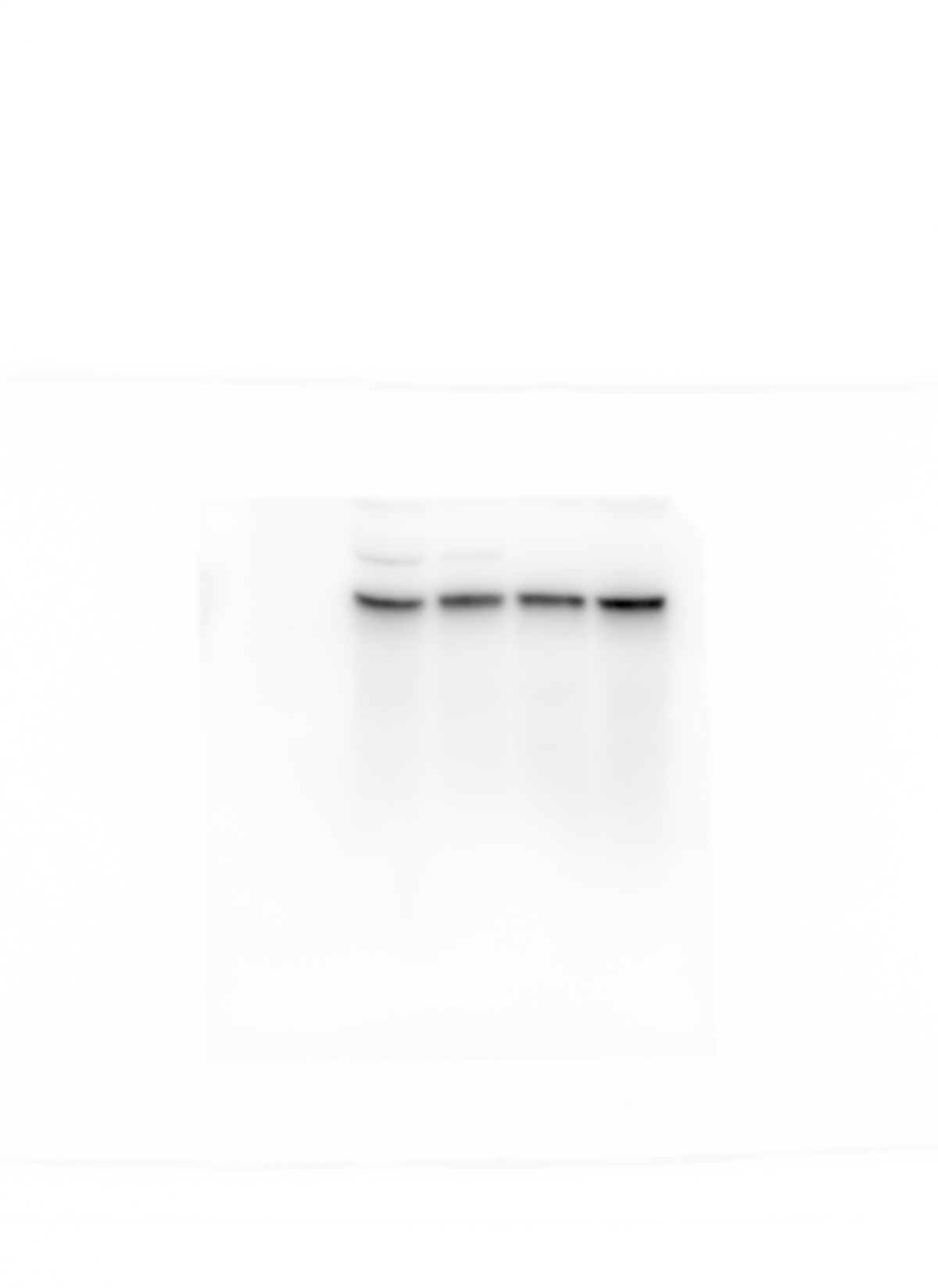

Supplement: Supplementary file 10 — Source data Fig. 5 [file 44318_2026_720_MOESM10_ESM.zip › SDfigure5/5C/total_eIF2a.tif]

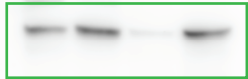

eIF2aP

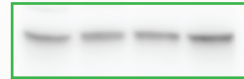

total eIF2a

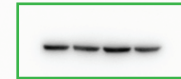

bAct

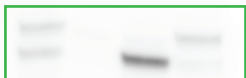

ZAP

Supplement: Supplementary file 10 — Source data Fig. 5 [file 44318_2026_720_MOESM10_ESM.zip › SDfigure5/5C/README.pdf]

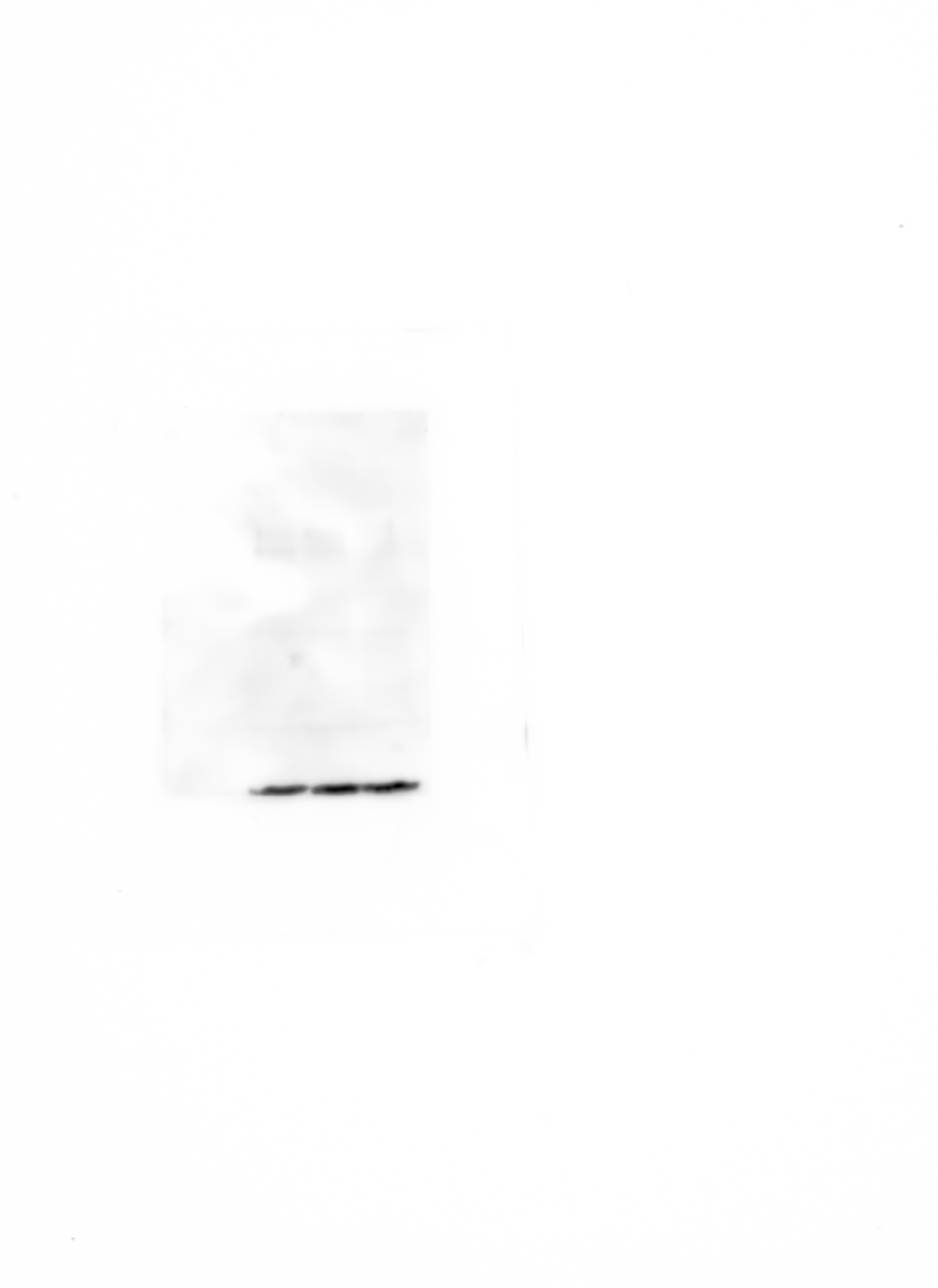

Supplement: Supplementary file 10 — Source data Fig. 5 [file 44318_2026_720_MOESM10_ESM.zip › SDfigure5/5B/bAct.tif]

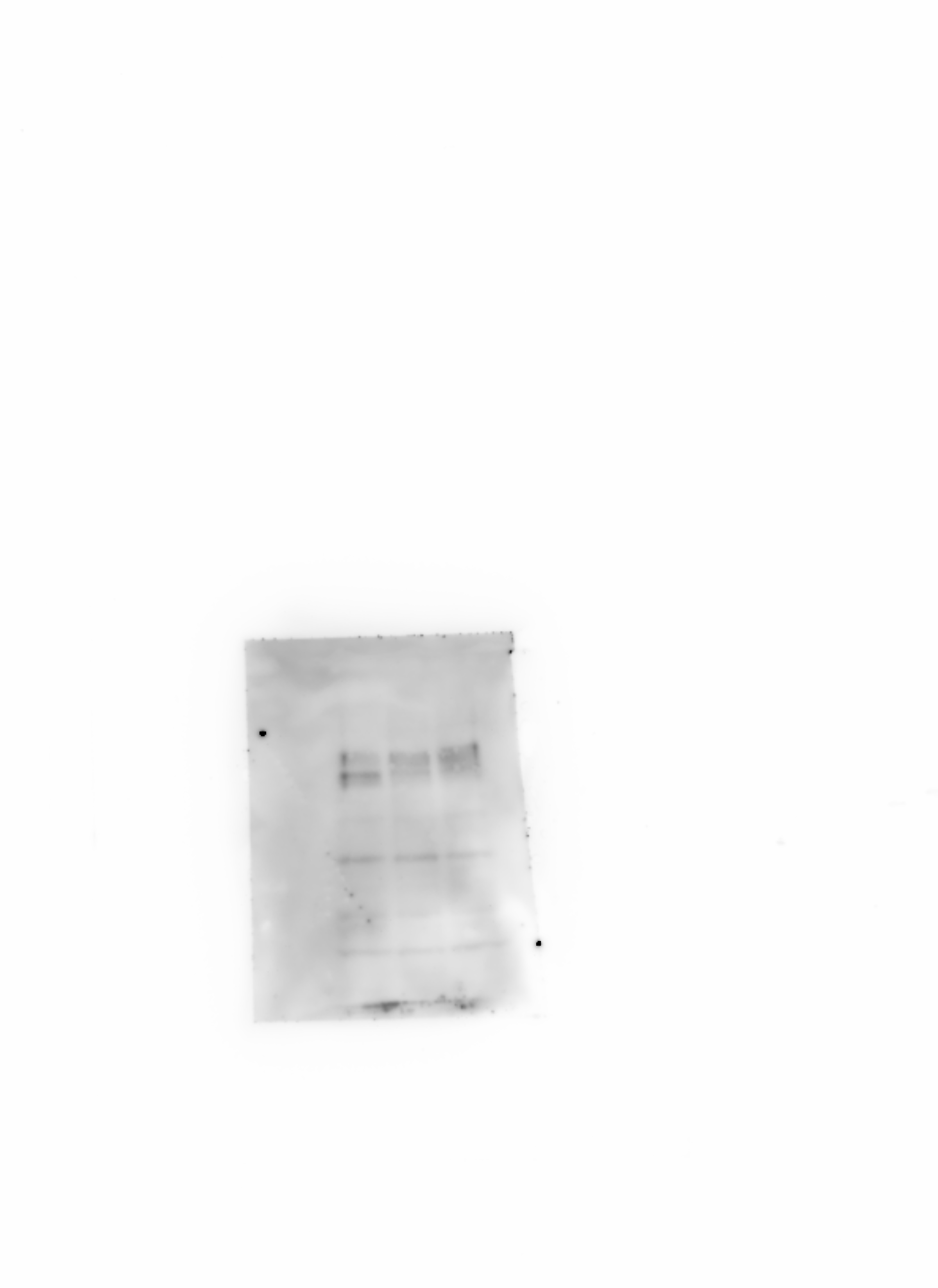

Supplement: Supplementary file 10 — Source data Fig. 5 [file 44318_2026_720_MOESM10_ESM.zip › SDfigure5/5B/PERK_Phostag.tif]

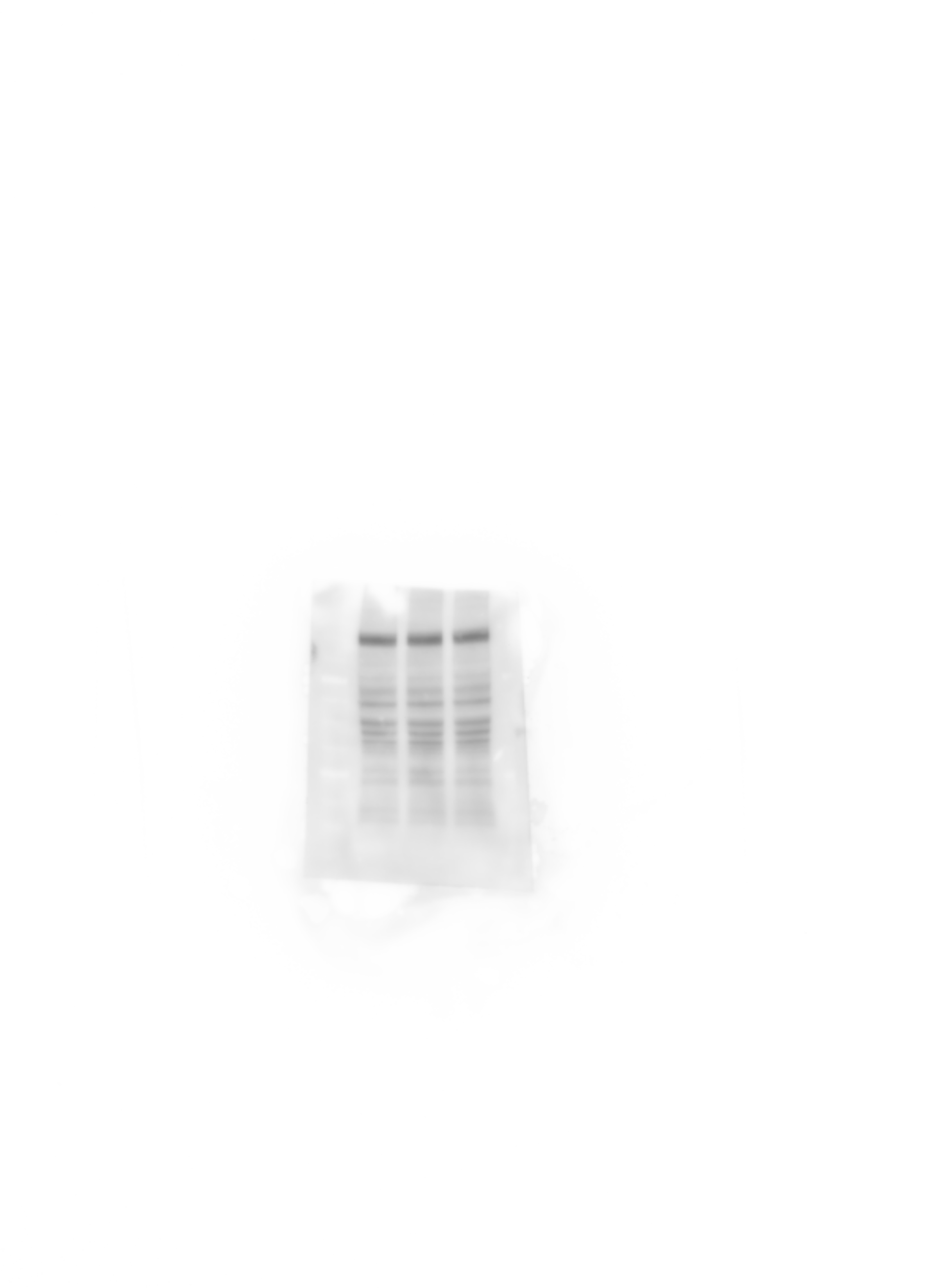

Supplement: Supplementary file 10 — Source data Fig. 5 [file 44318_2026_720_MOESM10_ESM.zip › SDfigure5/5B/PERK_SDS-PAGE.tif]

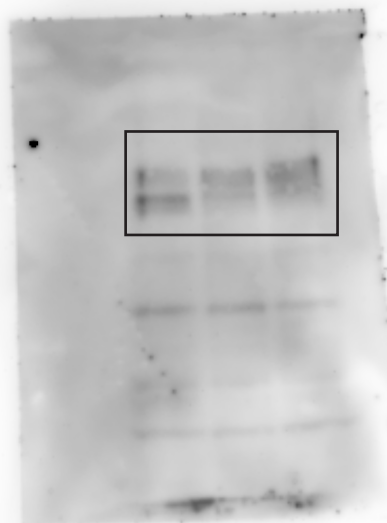

PERK

Phos-tag

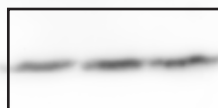

b-Act

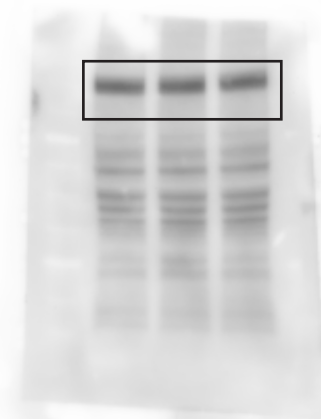

PERK  
SDS-PAGE

Supplement: Supplementary file 10 — Source data Fig. 5 [file 44318_2026_720_MOESM10_ESM.zip › SDfigure5/5B/README.pdf]
