# Supplementary material for: ZAP targets aberrant mRNA transcripts encoding proteins with defective signal peptides for degradation
Source: EMBO J. 2026 Mar 12;45(8):2638–65. doi: 10.1038/s44318-026-00720-4 (PMC13084044; doi:10.1038/s44318-026-00720-4)
Supplement: Supplementary file 11 — Expanded View Figures [file 44318_2026_720_MOESM11_ESM.pdf]

Expanded View Figures

Figure EV1. Establishing a reporter system to study the RAPP quality control pathway.

(**A**) RT-qPCR analysis of relative mCherry mRNA levels normalized to EGFP mRNA in HeLa cells transduced with the PPL-WT, Δ2 L, or Δ4 L reporter. Error bars indicate standard deviations (biological replicates, $n = 3$). Student's t test is indicated by asterisks. **$P < 0.01$. (**B**) Immunoblots for the RAPP reporters in HeLa cells. EGFP serves as an expression and loading control. SP*-mCherry denotes mCherry retaining an uncleaved signal peptide. (**C**) Representative immunofluorescence images of K562 cells expressing the PPL-WT reporter stained with anti-Calreticulin, anti-FALG (PPL-WT reporter), and anti-EGFP antibodies. Colocalization of calreticulin and the PPL-WT reporter is shown. Scale bar, 5 µm. (**D**) Flow cytometric analysis of monoclonal K562 cells expressing the PPL-WT or PPL-Δ2 L reporter. (**E**) Flow cytometric analysis of PPL-Δ2 L reporter cells transduced with sgRNA targeting mCherry compared to a control (Ctrl) sgRNA.

 © The Author(s)

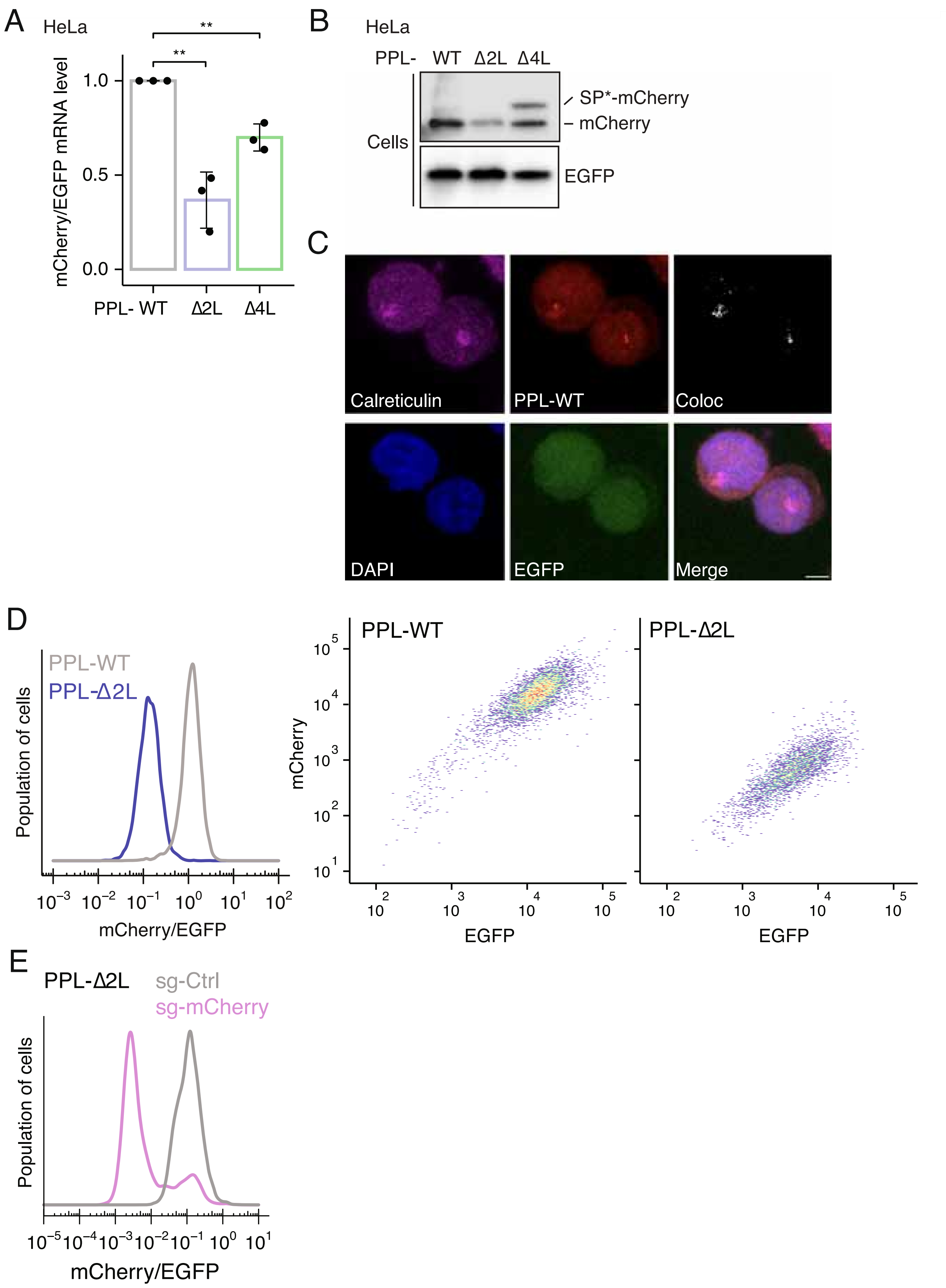
A
HeLa
**
**
mCherry/EGFP mRNA level
1.0
0.5
0.0
PPL- WT
Δ2L
Δ4L
B
HeLa
PPL- WT Δ2L Δ4L
Cells
SP*-mCherry
mCherry
EGFP
C
Calreticulin
PPL-WT
Coloc
DAPI
EGFP
Merge
D
PPL-WT
PPL-Δ2L
Population of cells
mCherry/EGFP
mCherry
EGFP
PPL-WT
PPL-Δ2L
E
PPL-Δ2L
sg-Ctrl
sg-mCherry
Population of cells
mCherry/EGFP

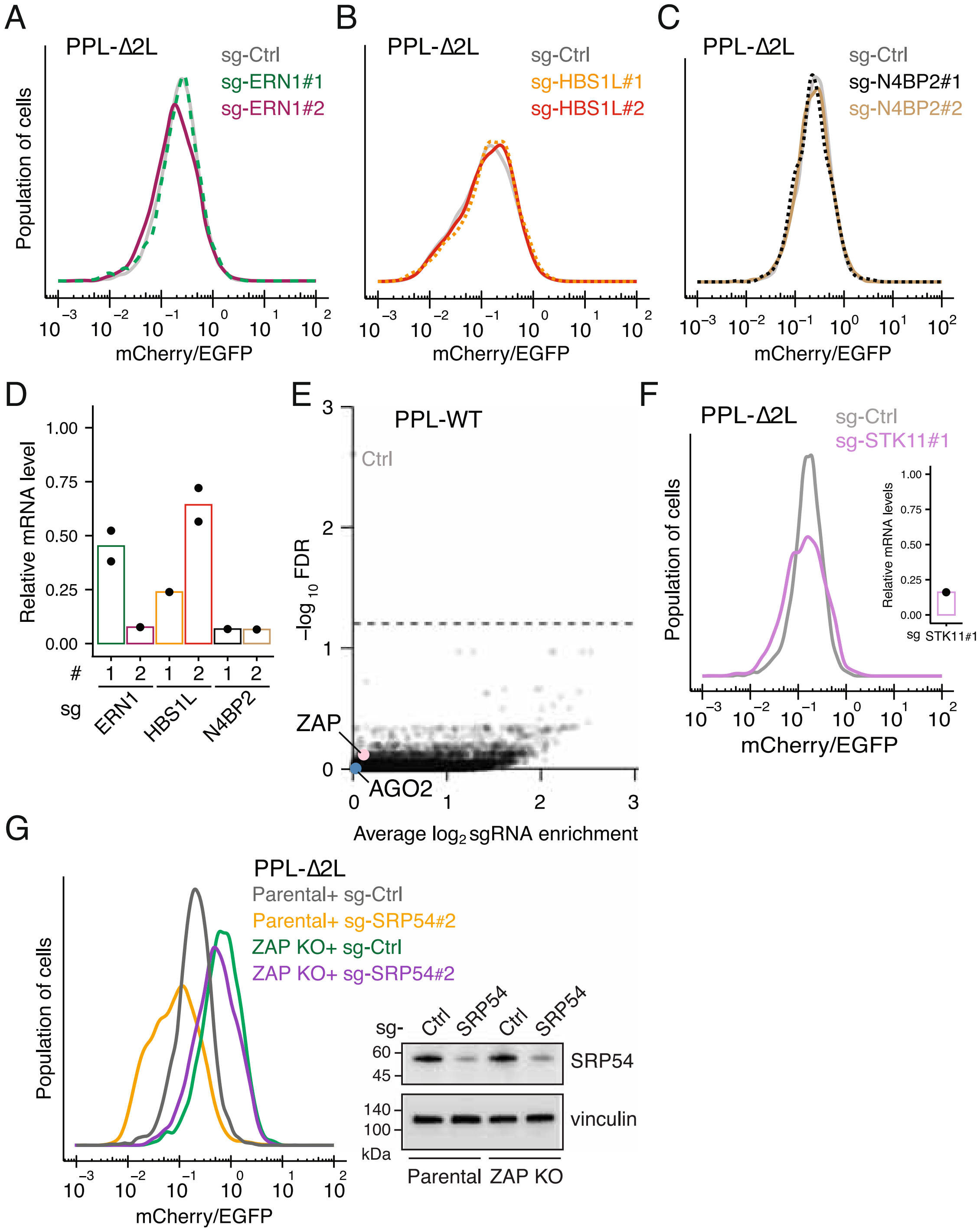

Figure EV2. A control genetic screen using the PPL-WT reporter.

(**A–C**) Flow cytometric analysis of PPL-Δ2 L reporter cells after transduction of sgRNAs targeting ERN1 (**A**), HBS1L (**B**), and N4BP2 (**C**). (**D**) RT-qPCR analysis of target gene expression corresponding to sgRNAs shown in (**A–C**). Each dot represents the mean of three technical replicates (biological replicates, $n \geq 1$). (**E**) A control FACS-based CRISPR screen using the PPL-WT reporter. Volcano plot showing effect size (average $\log_2$ fold change, *x* axis) versus false discovery rate (−log(FDR), y axis). AGO2 and ZAP are highlighted in blue and pink, respectively. (**F**) Flow cytometric analysis of PPL-Δ2 L reporter cells after sgRNA-mediated ablation of STK11 compared to a control sgRNA (left). Validation of STK11 knockdown by RT-qPCR (right). (**G**) Flow cytometric analysis of parental and ZAP KO cells expressing the PPL-Δ2 L reporter following depletion of SRP54 (left). Validation of SRP54 knockdown by immunoblotting (right).

© The Author(s)

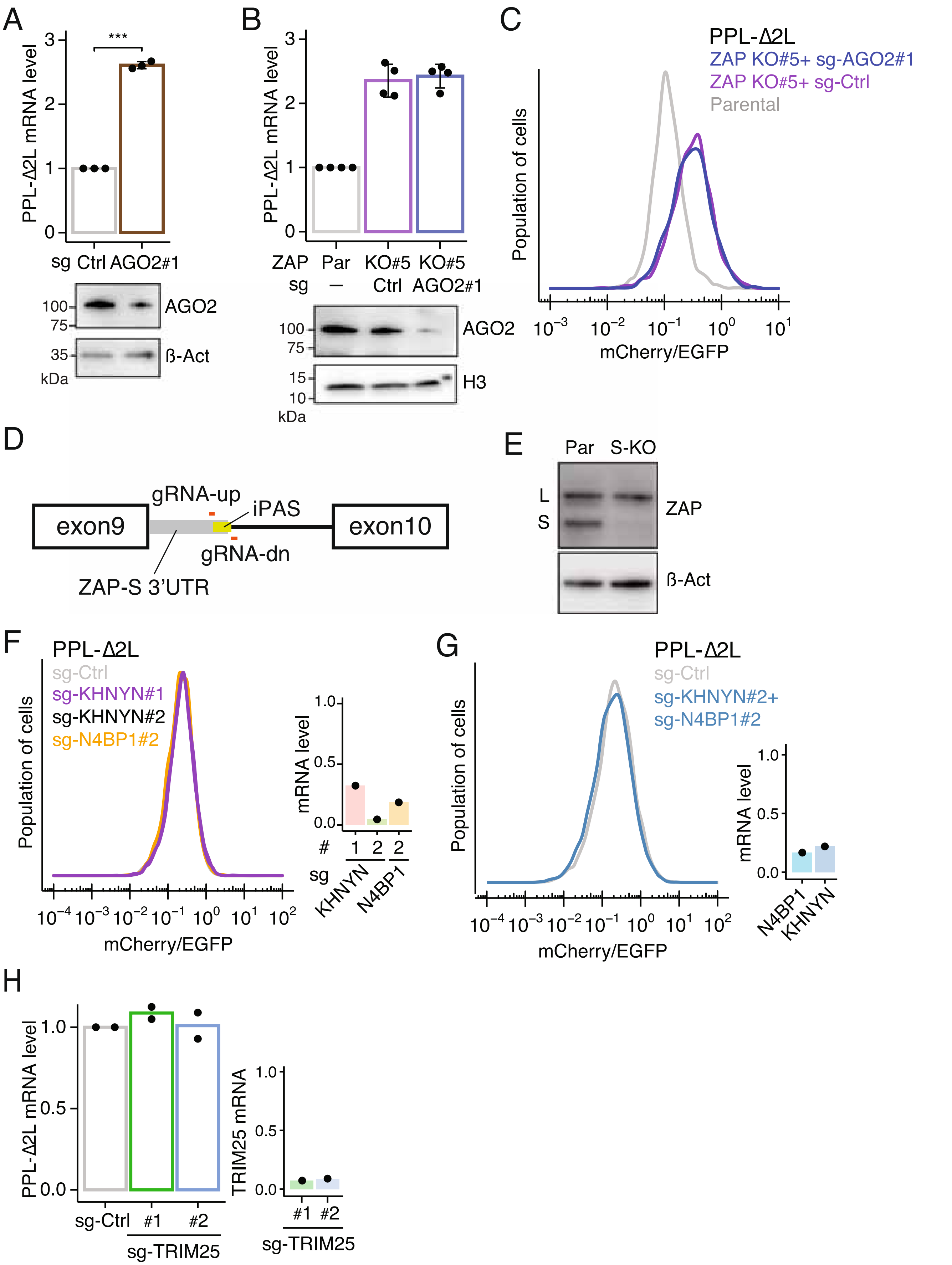
A
PPL-Δ2L mRNA level
***
sg Ctrl AGO2#1
100
75
AGO2
35
ß-Act
kDa
B
PPL-Δ2L mRNA level
ZAP Par KO#5 KO#5
sg — Ctrl AGO2#1
AGO2
H3
kDa
C
PPL-Δ2L
ZAP KO#5+ sg-AGO2#1
ZAP KO#5+ sg-Ctrl
Parental
Population of cells
mCherry/EGFP
D
exon9
gRNA-up
iPAS
exon10
gRNA-dn
ZAP-S 3'UTR
E
Par S-KO
L
S
ZAP
ß-Act
F
PPL-Δ2L
sg-Ctrl
sg-KHNYN#1
sg-KHNYN#2
sg-N4BP1#2
Population of cells
mCherry/EGFP
mRNA level
#
sg
KHNYN
N4BP1
G
PPL-Δ2L
sg-Ctrl
sg-KHNYN#2+
sg-N4BP1#2
Population of cells
mCherry/EGFP
mRNA level
N4BP1
KHNYN
H
PPL-Δ2L mRNA level
sg-Ctrl #1 #2
sg-TRIM25
TRIM25 mRNA
#1 #2
sg-TRIM25

Figure EV3. ZAP-S participates in the RAPP pathway.

(**A**) RT-qPCR assessment of PPL-Δ2 L reporter mRNA following Cas9-mediated AGO2 ablation. Error bars indicate standard deviations (biological replicates, $n = 3$) (top). Student's *t* test is indicated by asterisks. ***$P < 0.001$. Immunoblots for AGO2 (bottom). (**B**, **C**) RT-qPCR (**B**) and flow cytometric (**C**) analyses of PPL-Δ2 L reporter expression in parental and ZAP KO cells after sgRNA-mediated ablation of AGO2. Error bars indicate standard deviations (biological replicates, $n = 4$). (**D**) Schematic of ZAP exons 9 and 10. The 3′ UTR of ZAP-S isoform is indicated in grey, followed by an intronic polyadenylation signal (iPAS). A gRNA pair (gRNA-up and gRNA-dn) was utilized to remove the iPAS to knock out ZAP-S isoform. (**E**) Immunoblots of parental and a ZAP-S KO isogenic cell line. Two major ZAP isoforms are indicated. (**F**) Flow cytometric analysis of PPL-Δ2 L reporter cells following transduction of sgRNAs targeting N4BP2 or KHNYN compared to a control sgRNA (left). N4BP2 and KHNYN mRNA levels were quantified by RT-qPCR (right). (**G**) Similar to (**F**), PPL-Δ2 L reporter cells were transduced with sgRNAs targeting both N4BP2 and KHNYN (left). Efficiency of N4BP2/KHNYN double depletion was quantified by RT-qPCR (right). (**H**) RT-qPCR analysis of PPL-Δ2 L reporter mRNA levels following Cas9-mediated knockdown of TRIM25 compared to a control sgRNA (left) (biological replicates, $n = 2$). Knockdown efficiency of TRIM25 was assessed by RT-qPCR (right).

 © The Author(s)

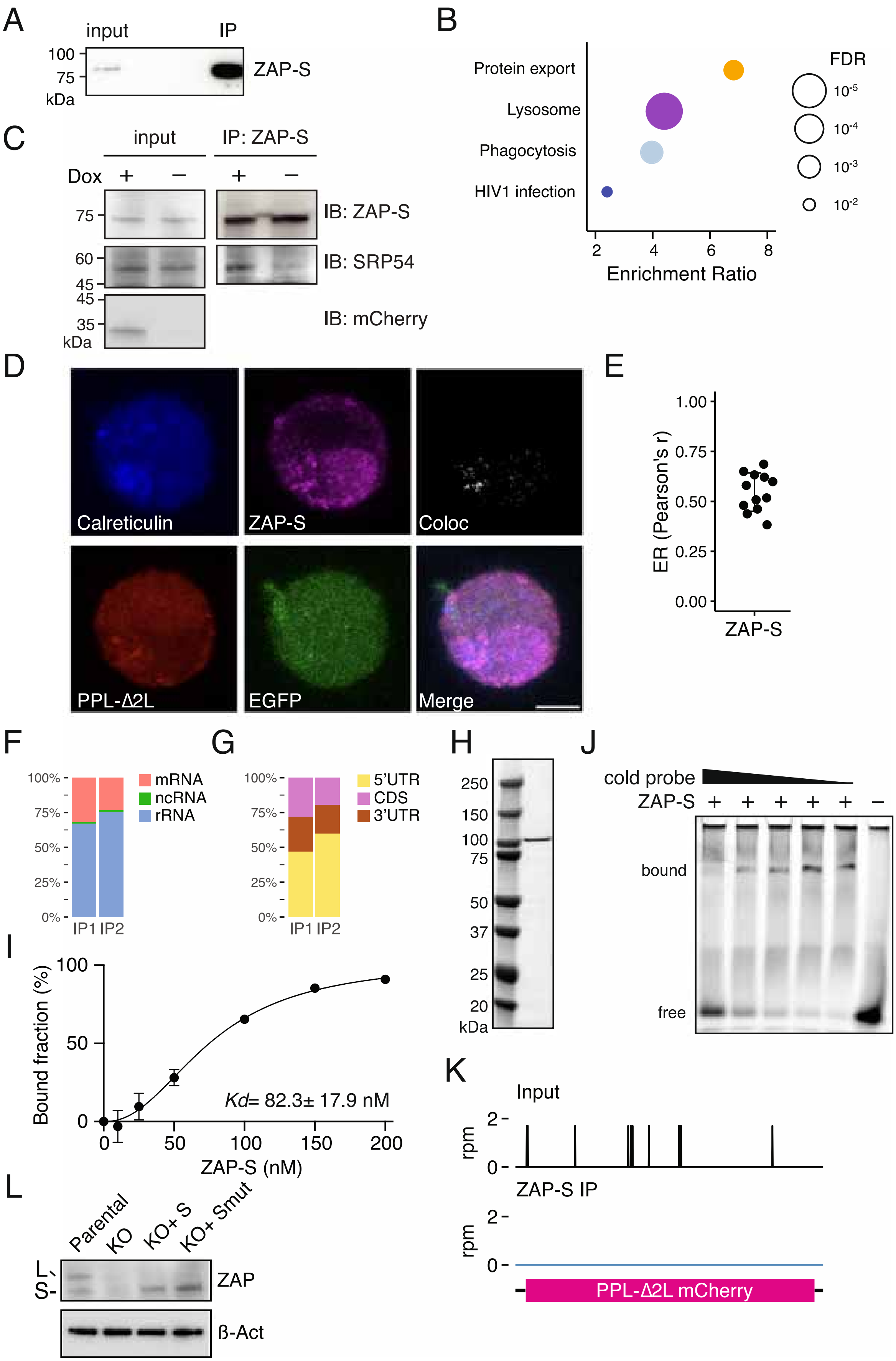

A
input
IP
100
75
kDa
ZAP-S
B
Protein export
Lysosome
Phagocytosis
HIV1 infection
FDR
Enrichment Ratio
C
input
IP: ZAP-S
Dox
IB: ZAP-S
IB: SRP54
IB: mCherry
D
Calreticulin
ZAP-S
Coloc
PPL-Δ2L
EGFP
Merge
E
ER (Pearson's r)
ZAP-S
F
mRNA
ncRNA
rRNA
IP1 IP2
G
5'UTR
CDS
3'UTR
IP1 IP2
H
J
cold probe
ZAP-S
bound
free
I
Bound fraction (%)
Kd= 82.3± 17.9 nM
ZAP-S (nM)
K
Input
rpm
ZAP-S IP
PPL-Δ2L mCherry
L
Parental
KO
KO+ S
KO+ Smut
ZAP
ß-Act

Figure EV4. ZAP-S facilitates RAPP through its interaction with the SRP.

(**A**) Immunoblot for HA-tagged ZAP-S immunoprecipitation. (**B**) Top four enriched biological processes identified from ZAP-S associated proteins in Fig. 4A. False discovery rate (FDR) is denoted by dot size. (**C**) Validation of the interaction between ZAP-S and SRP54 by immunoblotting. Expression of the PPL-Δ2 L reporter was induced by Dox (doxycycline). (**D**) Representative images of K562 cells expressing the PPL-Δ2 L reporter stained with anti-Calreticulin, anti-HA (ZAP-S), anti-FLAG (PPL-Δ2 L reporter), and anti-GFP antibodies. Colocalization of calreticulin and ZAP-S is shown. Scale bar, 5 µm. (**E**) Quantification of ZAP-S colocalization with the calreticulin-positive pixels using Pearson's r values. Error bars indicate standard deviations ($n = 12$). (**F**) Composition of reproducible ZAP-S crosslink sites detected in two IP replicates. (**G**) Distribution of ZAP-S crosslinked reads from (**F**) across mRNAs (5′UTR, CDS, and 3′UTR). (**H**) Coomassie staining of purified recombinant ZAP-S protein. (**I**) EMSA experiment of ZAP-S binding to the 7SL RNA probe. *Kd* is indicated ± standard deviations ($n = 3$). (**J**) Competition EMSA showing specific ZAP-S binding to the 7SL RNA probe. Increasing amounts of unlabeled (cold) 7SL RNA probe were added to compete with labeled 7SL RNA probe. (**K**) Gene model of the PPL-Δ2 L reporter showing reads from input (black) and ZAP-S IP (blue) samples. (**L**) Immunoblot for ZAP KO cells rescued with ZAP-S WT or K107A/Y108A mutant.

 © The Author(s)

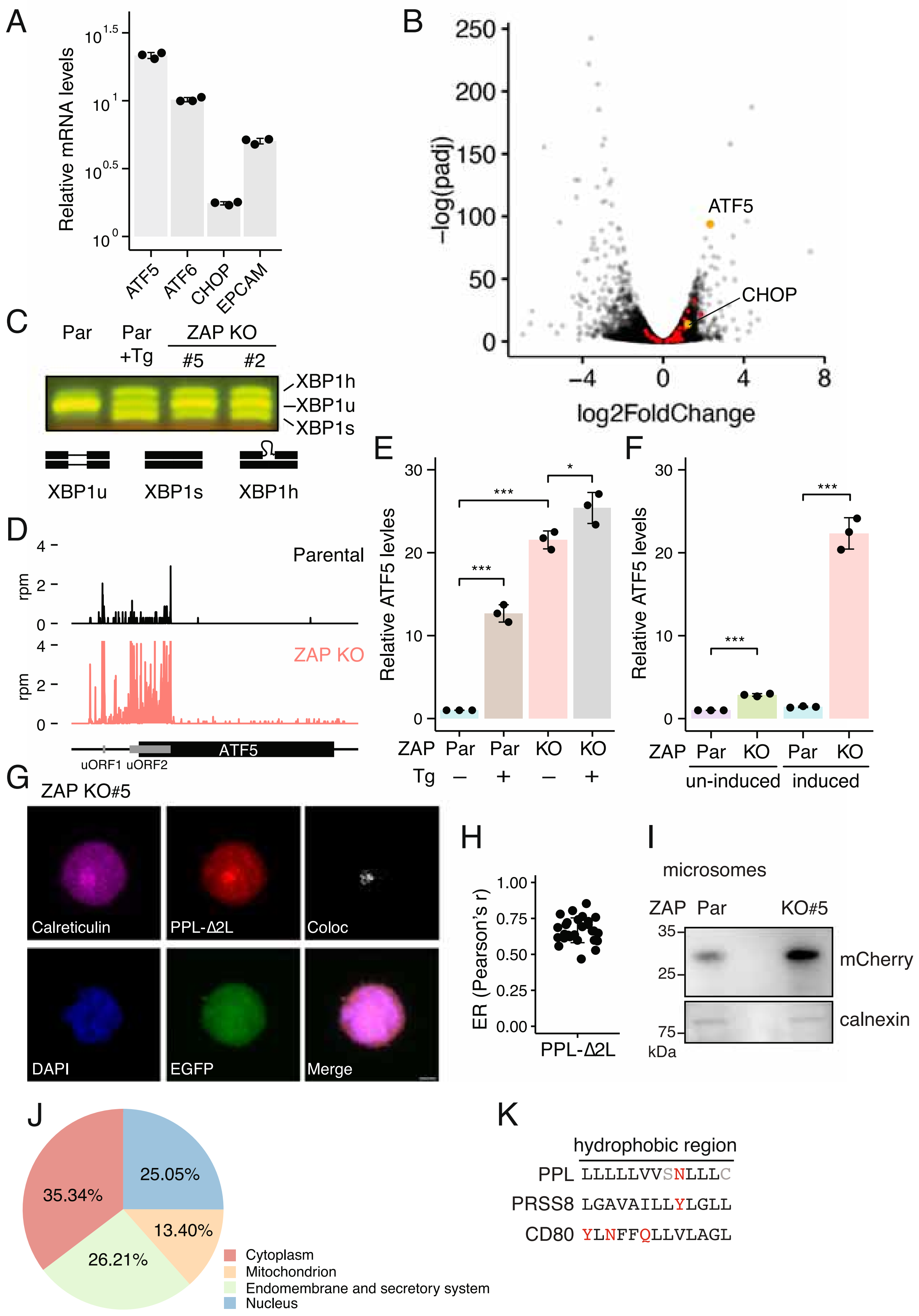
A
Relative mRNA levels
ATF5
ATF6
CHOP
EPCAM
B
ATF5
CHOP
–log(padj)
log2FoldChange
C
Par
Par +Tg
ZAP KO
#5
#2
XBP1h
XBP1u
XBP1s
XBP1u
XBP1s
XBP1h
D
Parental
ZAP KO
rpm
ATF5
uORF1 uORF2
E
Relative ATF5 levles
ZAP Par Par KO KO
Tg – + – +
F
Relative ATF5 levels
ZAP Par KO Par KO
un-induced induced
G
ZAP KO#5
Calreticulin
PPL-Δ2L
Coloc
DAPI
EGFP
Merge
H
ER (Pearson's r)
PPL-Δ2L
I
microsomes
ZAP Par KO#5
mCherry
calnexin
kDa
J
35.34%
25.05%
13.40%
26.21%
Cytoplasm
Mitochondrion
Endomembrane and secretory system
Nucleus
K
hydrophobic region
PPL LLLLLVVSNLLLC
PRSS8 LGAVAILLYLGLL
CD80 YLNFFQLLVLAGL

© The Author(s)

Figure EV5. Loss-of-function of ZAP induces the UPR.

(**A**) Validation of RNA-seq results in ZAP KO cells by RT-qPCR. ATF6, ATF5, CHOP, and EPCAM (a representative secretory protein) mRNA levels are elevated in ZAP KO cells. Error bars indicate standard deviations (biological replicates, $n = 3$). (**B**) Volcano plot of RNA-seq results comparing ZAP KO to parental cells. ATF5 and CHOP are shown in orange and CHOP-dependent genes in red (biological replicates, $n = 2$). (**C**) RT-PCR analysis of XBP1 splicing in parental and two isogenic ZAP KO lines. Thapsigargin (Tg) treatment (2 µM for 24 h) was used as a positive control for UPR activation. (**D**) Ribosome footprint density across ATF5 from parental and ZAP KO cells. uORFs are annotated in grey. (**E**) RT-qPCR analysis of ATF5 expression levels in parental and ZAP KO cells after thapsigargin (Tg) treatment (2 µM for 24 h). Cells were cultured in the presence of doxycycline to induce the PPL-Δ2 L reporter. Error bars indicate standard deviations (biological replicates, $n = 3$). Student's *t* test is indicated by asterisks. *$P < 0.05$; ***$P < 0.001$. (**F**) RT-qPCR analysis of ATF5 expression levels in parental and ZAP KO cells with or without induction of the PPL-Δ2 L reporter by doxycycline. Error bars indicate standard deviations (biological replicates, $n = 3$). Student's *t* test is indicated by asterisks. ***$P < 0.001$. (**G**) Representative images of K562 ZAP KO cells expressing the PPL-Δ2 L reporter stained with anti-Calreticulin, anti-FLAG (PPL-Δ2 L reporter), and anti-GFP antibodies. Colocalization of calreticulin and the PPL-2L reporter is shown. Scale bar, 5 µm. (**H**) Quantification of PPL-Δ2 L colocalization with the calreticulin-positive pixels using Pearson's r values ($n = 27$). (**I**) Immunoblots for the PPL-Δ2 L reporter from parental and ZAP KO microsomal fractions. Calnexin serves as an ER marker. (**J**) BUSCA subcellular localization analysis of upregulated genes in ZAP KO cells. Endomembrane and secretory system includes extracellular space (GO:0005615), endomembrane system (GO:0012505), plasma membrane (GO:0005886), and organelle membrane (GO:0031090). (**K**) Hydrophobic regions of PPL, PRSS8, and CD80 signal peptides. Hydrophobic residues are shown in black, disfavored amino acids in red, and all other residues in grey.

 © The Author(s)
